# Ross River virus transmission, infection, and disease: two and a half decades of research progress—an updated cross-disciplinary review

**DOI:** 10.1128/cmr.00099-25

**Published:** 2026-06-04

**Authors:** E. B. Skinner, D. Harley, A. F. van den Hurk, A. Michie, M. Aubry, B. M. Martin, D. J. Rawle

**Affiliations:** 1 Frazer Institute, Faculty of Health, Medicine and Behavioural Sciences, The University of Queensland, Brisbane, Queensland, Australia; 2 Metro North Public Health Unit, Metro North Hospital and Health Service, Brisbane, Queensland, Australia; 3 Public Health Virology, Public and Environmental Health Reference Laboratories, Department of Health, Queensland Government, Coopers Plains, Queensland, Australia; 4 The Kirby Institute, University of New South Wales, Kensington, New South Wales, Australia; 5 Virology Research Laboratory, Prince of Wales Hospital, Randwick, New South Wales, Australia; 6 Laboratory of Research on Emerging Viral Diseases, Institut Louis Malardé 92966, Papeete, Tahiti, French Polynesia; 7 Infection and Inflammation Department, QIMR Berghofer, Brisbane, Queensland, Australia; 8 School of Chemistry and Molecular Biosciences, University of Queensland 198110, St. Lucia, Queensland, Australia; 9 GVN Center of Excellence, Australian Infectious Disease Research Centre, Brisbane, Queensland, Australia; Rush University Medical Center, Chicago, Illinois, USA; The University of Western Australia, Perth, Australia

**Keywords:** arbovirus, phylogenetics, epidemiology, vector control, ecology, reservoir host, alphavirus

## Abstract

Ross River virus (RRV) causes the most mosquito-borne disease notifications in Australia and a considerable burden of non-fatal, yet frequently prolonged rheumatic illness across the Australia-Pacific region, reflected in thousands of notifications each year and notable economic and quality-of-life losses. Research on RRV spans multiple disciplines, encompassing viral genomics, immunopathology, transmission ecology, epidemiology, entomology, and environmental science. This review synthesizes two decades of cross-disciplinary investigations to present an integrated perspective on the biological, clinical, ecological, and environmental dimensions of RRV infection. By consolidating findings from diverse fields, the review enhances understanding of the complex factors influencing RRV transmission and disease outcomes throughout its endemic range.

## INTRODUCTION

Ross River virus (RRV) is a mosquito-borne alphavirus of the family *Togaviridae* that causes the most commonly notified mosquito-borne disease in Australia (1). It is one of more than 70 arboviruses circulating in the country, but among the few that are nationally notifiable (2). Although not fatal, RRV disease imposes a substantial health and economic burden. Each year, the combined illness impacts add up to nearly 188 years lived with disability (YLDs) (3), which represents the total equivalent time spent by all affected individuals living in less-than-full health due to RRV, with direct economic costs estimated at $15 million per year (4). Beyond Australia, RRV is endemic in several Pacific Island Countries and Territories (PICTs) based on serological surveys, genomic divergence from Australian strains, and clinical cases in returning travelers (1, 5, 6). Given RRV’s ability to spread in immunologically naive populations and the involvement of many vectors and hosts in its transmission, it could, like chikungunya virus (CHIKV) and Zika virus (ZIKV), become a global arboviral threat (7).

Ross River virus presents considerable challenges for prevention, diagnosis, and management. Transmission involves multiple mosquito vectors and vertebrate reservoir hosts, with patterns varying geographically across endemic regions (8). The ecological complexity complicates outbreak prediction and prevention efforts, which currently rely primarily on mosquito control programs and personal protective measures against mosquito bites (9). A brief viraemic period and reliance on serological testing, with antibodies cross-reacting with other alphaviruses, including Barmah Forest virus (BFV), similar clinically and in distribution, make it challenging to gain definitive laboratory evidence of infection (10). No licensed vaccine or targeted antiviral therapy currently exists, leaving disease management limited to symptomatic treatment. These challenges are compounded by the impact of climate change, urbanization, human behavior, and land-use modification, which are altering vector and host distributions and abundance, while increasing human contact with enzootic transmission cycles (3, 11–13).

Addressing these challenges has driven substantial research progress in the two decades since Harley and colleagues’ landmark 2001 review (14). Genomic and phylogenetic studies have mapped viral evolution and geographic spread; research has revealed mechanisms of infection and inflammatory pathogenesis; ecological and epidemiological investigations have expanded knowledge of hosts, vectors, and environmental drivers of transmission; and modeling and clinical research have improved outbreak forecasting, refined disease burden estimates, and stimulated progress in vaccine and therapeutic development. The current review aims to synthesize these advances to provide an updated, cross-disciplinary perspective on RRV, highlighting both recent progress and the remaining scientific and public health priorities.

## HISTORY

### Australia perspective

**Fig 1:**
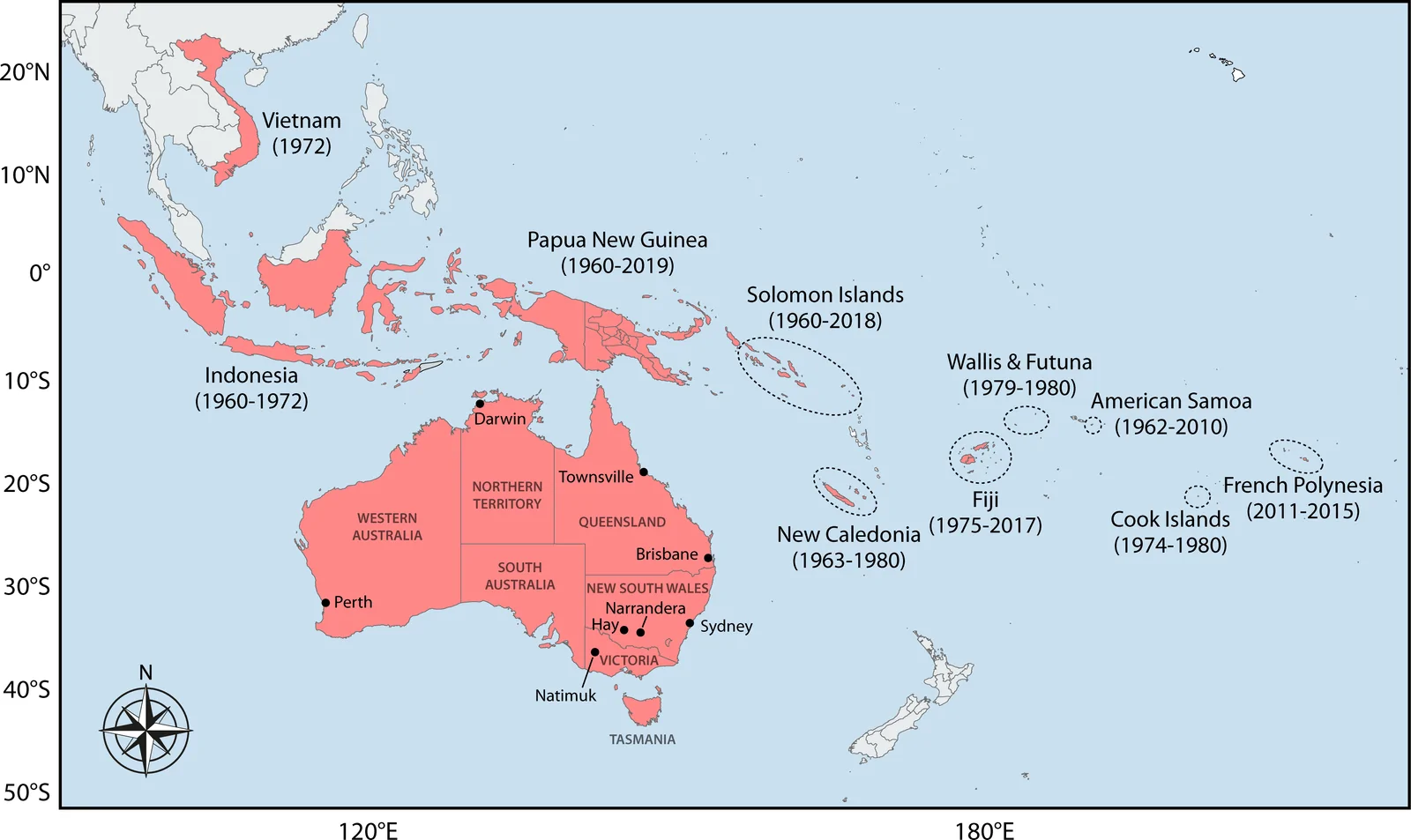
Map showing countries, states, territories, cities, and towns discussed within the text. Countries in pink are those with reported localized evidence of RRV transmission, with the year ranges being the years of investigation, not active transmission. The base map was generated in R; the code uses rnaturalearth, specifically the ne_countries() function from the rnaturalearth and rnaturalearthdata R packages.

Ross River virus has likely circulated long before the first documented cases, although historical records, particularly for Indigenous populations, are limited. The earliest recorded accounts of RRV-like illness, known as “epidemic polyarthritis,” predate the isolation of the virus. In 1886, an outbreak of 30 cases of polyarthritis, fever, and rash was reported in Natimuk, Victoria (VIC), which may be the earliest documented outbreak of RRV (15–17) (Fig. 1). At the time, the outbreak was thought to be dengue or typhoid (due to overlapping clinical symptoms); however, given the absence of dengue virus vectors in this region, it has since been re-evaluated as a Ross River virus disease outbreak (16, 18). More than 30 years later, another “unusual epidemic” in 1928 in Narrandera and Hay, two towns in New South Wales (NSW), affected more than 100 people (16, 19–21) (Fig. 1). The outbreak was similarly attributed to dengue, with speculation that it originated from a traveler arriving from Queensland (QLD) (21); however, the absence of dengue virus vectors in the region again made this unlikely (22), and it has since been considered an early outbreak of RRV or BFV, a related arthritic alphavirus.

During 1941–1942, army medical officers in northern Australia noted increasing numbers of soldiers reporting symptoms of pain and stiffness of joints, headache, rash, and sometimes mild fever of unknown origin (23). The clinical features for the diagnosis of “acute polyarthritis” were characterized following an outbreak affecting 51 people in a military camp 300 km south of Darwin, Northern Territory (23). Over the decade that followed, recognition of the syndrome expanded beyond military populations and northern regions of Australia. In 1956, a widespread outbreak of epidemic polyarthritis occurred in the Murray Valley region of southern Australia (24). These epidemics were then linked to preceding heavy rainfall and flooding, with the implication that insect vectors transmitted the pathogen (25). By 1960, Shope and Anderson (26) proposed a viral etiology similar, but not identical, to the arthropod-borne virus CHIKV, which was causing outbreaks in Africa.

The first isolate of RRV was recovered from *Aedes vigilax* mosquitoes collected from beside the Ross River in Townsville, QLD, in 1959 (27) (Fig. 1). This prototype strain, designated T48, was identified as an antigenic group A arbovirus and a member of the *Alphavirus* genus (family *Togaviridae*), related to other arthritogenic viruses, such as CHIKV. While this isolation confirmed the presence of a previously undocumented mosquito-borne alphavirus, it did not demonstrate that RRV caused human disease, as no human isolates or direct clinical correlation were available. The first human isolate of RRV was obtained from a 7-year-old Indigenous Australian presenting with fever and headache in 1971 (28). This confirmed that RRV could infect humans, but the absence of arthralgia or arthritis in this case did not confirm the link between RRV and epidemic polyarthritis. Serological studies in the 1960s and early 1970s linked RRV to human disease by demonstrating significant antibody titers to the RRV antigen in the serum of people with historical epidemic polyarthritis (26, 27, 29, 30).

In 1985, RRV was finally isolated from an Australian patient presenting with typical symptoms of RRV infection, definitively confirming RRV as the causative agent of epidemic polyarthritis in Australia (31). Following this, a series of major outbreaks occurred across multiple Australian states during the 1980s and early 1990s. The increasing public health burden of these outbreaks led to RRV becoming a nationally notifiable disease in 1991, establishing standardized reporting across Australia, although surveillance systems and case definitions have changed several times since then.

### Pacific perspective

The first decisive confirmation of RRV as the causative agent of epidemic polyarthritis came during the 1979–1980 Pacific epidemic, which affected Fiji, American Samoa, Cook Islands, Tonga, New Caledonia, Wallis & Futuna, and Vanuatu (32–36). During this epidemic, RRV was successfully isolated from the serum of approximately half of the patients in the Cook Islands with serologically proven polyarthritis, which was in contrast to the Australian experience, where the virus had never been isolated from patients with polyarthritis (32). RRV was also isolated from symptomatic polyarthritis patients in Fiji, American Samoa, New Caledonia, and Wallis & Futuna during this period (33–36) (Fig. 1). Serological surveys conducted before this epidemic using sera from populations in Vanuatu, New Caledonia, American Samoa, Palau, and the Cook Islands found no RRV antibodies, indicating that the virus had not previously circulated in these countries (32, 33, 35–37) (Table 1; Fig. 1). In contrast, evidence of RRV transmission before the epidemic was apparent in parts of the Pacific, with seropositivity rates up to 64% in Papua New Guinea, 17% in the Solomon Islands (37), and 13% in Fiji (34), and RRV antibodies were also found among some localities in Indonesia and South Vietnam (37). The epidemic caused dramatic seroprevalence increases in previously naive populations: from 0% to 69% in the Cook Islands (32) and from 0% to 44% in American Samoa (33), while Fiji’s seroprevalence increased from 13% to 92% (34) (Table 1).

**TABLE 1:** Temporal, serological, phylogenetic, and clinical evidence of RRV transmission in the Asia-Pacific region outside Australia, 1960–2019

Country/territory	Sample yr	Study population	Evidence type	Key finding	Reference
Indonesia	1960–1972	General populations, adolescents, adults, and medical students across multiple localities	Serological testing by plaque reduction neutralization assay	0%–95% (102/107) seroprevalence across localities	B. Tesh et al. (37)
South Vietnam	1972	General population	Serological testing by plaque reduction neutralization assay	1% (1/130) seroprevalence	B. Tesh et al. (37)
Papua New Guinea	1960–1969	General populations, children, and adults across multiple localities	Serological testing by plaque reduction neutralization assay	0% (0/33) to 64% (46/72) seroprevalence across localities	B. Tesh et al. (37)
	1980–1981	Port Moresby residents	Clinical cases with serological diagnosis using microtitre neutralization assay	3 confirmed and 3 probable RRV-associated polyarthritis cases among 24 tested	M. Scrimgeour et al. (38)
	1991	Southern Highlands Province residents	Serological testing by ELISA	59% (34/58) seroprevalence with age-related increase	Hii et al. (39)
	1997	*An. farauti* complex mosquitoes	Viral isolation and phylogenetics	RRV isolated; independent evolution from Australian lineages	Michie et al. (6), C. A. Johansen et al. (40)
	2019	Military personnel	Serological testing by micro-neutralization assay	56.4% (115/204) seroprevalence overall; 46.7% (49/105) in those born after 1982	G. Kizu et al. (41)
Solomon Islands	1960–1972	General populations and adults across multiple localities	Serological testing by plaque reduction neutralization assay	0% (0/32) to 17% (28/163) seroprevalence across localities	B. Tesh et al. (37)
	2018	General population aged ≥ 5 years	Serological testing by ELISA	31% (321/1021) seroprevalence with age-related increase	L. Russell et al. (42)
American Samoa	1962	Adults	Serological testing by plaque reduction neutralization assay	0% (0/30) seroprevalence	B. Tesh et al. (37)
	1972	Adult residents	Serological testing by plaque reduction neutralization assay	0% (0/100) seroprevalence	B. Tesh et al. (33)
	1979	Hospital and clinic patients	Serological testing by plaque reduction neutralization assay; Viral isolation	43.8% of 393 samples tested seropositive near epidemic end; RRV isolated from 1 patient with polyarthritis	B. Tesh et al. (33)
	2010	Adult population	Serological testing by ELISA	74% (145/196) seroprevalence overall; 75%–80% in those born 1985–1990; 45% in those born 1991–1993	Lau et al. (5)
Vanuatu	1963–1972	General populations and adults across multiple localities	Serological testing by plaque reduction neutralization assay	0% seroprevalence	B. Tesh et al. (37)
New Caledonia	1963	General population	Serological testing by plaque reduction neutralization assay	0% (0/72) seroprevalence	B. Tesh et al. (37)
	1979–1980	Polyarthritis patients	Viral isolation	RRV isolated from patients with polyarthritis	Fauran et al. (35), G. Panon et al. (36)
Wallis & Futuna	1979–1980	Polyarthritis patients	Viral isolation	RRV isolated from patients with polyarthritis	Fauran et al. (35)
Palau	1961 & 1974	General population	Serological testing by plaque reduction neutralization assay	0% (0/104) seroprevalence	B. Tesh et al. (37)
Cook Islands	1974	Adults ≥ 40 years	Serological testing by hemagglutination-inhibition and plaque reduction neutralization assays	0% (0/112) seroprevalence	Rosen et al. (32)
	1980	Adults	Serological testing by hemagglutination-inhibition assay; viral isolation	69% (68/99) seroprevalence; RRV isolated from 49 patients with acute infection	Rosen et al. (32)
Fiji	1975	Participants of a hepatitis survey	Serological testing by hemagglutination-inhibition assay	13% (30/225) seroprevalence	Aaskov et al. (34)
	1979–1980	Residents of some communities	Serological testing by hemagglutination-inhibition assay; Viral isolation	92% (386/418) seroprevalence; RRV isolated from 1 patient with polyarthritis	Aaskov et al. (34)
	1999	German traveler	Clinical case with serological diagnosis using neutralization assay	RRV infection confirmed in 1 returning traveler	Pröll et al. (43)
	2003–2004	Canadian travelers	Clinical cases with serological diagnosis using plaque reduction neutralization assay	RRV infection confirmed in two returning travelers	Klapsing et al. (44)
	2005	Suspected dengue patients	Serological testing by focus reduction neutralization assay	High-titer neutralizing RRV antibodies indicating recent infection in 53.6% of 52 samples tested	M. Ngwe Tun et al. (45)
	1997–2009	New Zealand travelers	Clinical cases with serological diagnosis by RRV IgM detection	RRV infection confirmed in 5 returning travelers	Lau et al. (46)
	2013	Cohort study participants initially recruited for a community-based serosurvey for leptospirosis and typhoid	Serological testing by microsphere immunoassay	46.5% (362/778) seroprevalence	Aubry et al. (47)
	2015	Cohort study participants	Serological testing by microsphere immunoassay	37.2% (124/333) seroprevalence; 10.9% (21/192) seroconverted since 2013	Aubry et al. (47)
	2017	Cohort study participants	Serological testing by microsphere immunoassay	39.1% (125/320) seroprevalence; 8% (16/200) seroconverted since 2015	Aubry et al. (48)
French Polynesia	2011–2013	Adult blood donors	Serological testing by ELISA	34.4% (204/593) seroprevalence overall with age-related increase	Aubry et al. (49)
	2014	General population	Serological testing by ELISA	35% (68/196) seroprevalence overall; 28% (23/81) in those born/arrived from 1982; 17% (3/18) in those born/arrived after 2000	Aubry et al. (50)
	2014	Schoolchildren aged 6-16 years	Serological testing by ELISA	1% (6/476) seroprevalence	Aubry et al. (50)
	2015	General population	Serological testing by microsphere immunoassay	18% (123/700) seroprevalence overall; 15% (46/303) in those born/arrived from 1982; 0% (0/54) in those born/arrived after 2000	Aubry et al. (50)

Although no RRV outbreaks have been reported in the PICTs since 1980, multiple lines of evidence support silent endemic circulation of RRV in the region over the subsequent decades (Table 1). Serological evidence from French Polynesia illustrates this cryptic transmission of RRV beyond Australia (Fig. 1). No cases were detected during intensified surveillance in the early 1980s (49), and subsequently, serological surveys from 2011 to 2015 revealed that RRV had circulated cryptically for decades. Age-stratified antibody patterns indicated continuous low-level transmission among residents born after the Pacific epidemic (49, 50), although declining seropositivity in younger individuals suggests increasingly sporadic transmission since 2000 (50) (Table 1). Similarly, in Fiji, longitudinal cohort studies documented active seroconversion and age-related increases in antibody prevalence between 2013 and 2017 (47, 48); in American Samoa, high seroprevalence persists among individuals born a decade after the epidemic (5); and in Papua New Guinea and Solomon Islands, age-stratified seroprevalence patterns continue to indicate ongoing endemic transmission (39, 41, 42) (Table 1).

Genomic and clinical evidence further confirm endemic persistence. Analysis of an RRV isolate recovered from *Anopheles farauti sensu lato* (*s.l*.) in Papua New Guinea in 1997 demonstrated that the virus had evolved independently from Australian lineages since its likely introduction during the 1979–1980 epidemic, indicating sustained local circulation (6). Clinical and serological evidence of RRV infections in international travelers returning from the Pacific provides direct confirmation of active transmission, including cases from Fiji documented between 1997 and 2009 (43, 44, 46) (Table 1). These travelers served as sentinels for ongoing transmission that went undetected by local surveillance systems.

The persistence of RRV transmission in PICTs for decades without detection reflects both favorable ecological conditions and limited surveillance capacity. The Pacific region provides suitable conditions for endemic RRV circulation: competent mosquito vectors are widely distributed (51), and serological evidence has demonstrated that non-human vertebrates are capable of maintaining transmission and are naturally exposed to RRV (52). Simultaneously, several factors have limited detection: many PICTs lack laboratory infrastructure for RRV diagnosis (46); most infections are asymptomatic or cause only mild symptoms that do not prompt medical attention (49); and symptomatic cases are frequently misdiagnosed as dengue or other arboviral diseases due to overlapping clinical presentations (42, 46). This combination has allowed RRV to circulate cryptically across the Pacific for over four decades, with transmission rates varying between the PICTs through time. The 1979–1980 Pacific epidemic demonstrated RRV’s epidemic potential, and the recent global spread of other arboviruses such as CHIKV and ZIKV suggests that RRV remains a threat for future pandemic emergence (7).

## RRV EPIDEMIOLOGY IN AUSTRALIA

### Economic and public health burden

Ross River virus imposes a substantial economic and public health burden on Australia through direct medical costs, productivity losses, and extensive investment in prevention and control programs. The disease burden has recently been quantified using YLDs, which account for the morbidity associated with RRV infection rather than mortality, as RRV-related deaths have not been reported in Australia. Analysis of Australian Institute of Health and Welfare (AIHW) Burden of Disease data from 2003 to 2018 reveals that RRV infections result in a cumulative loss equivalent to nearly 188 years of healthy life annually due to morbidity (3). The direct economic costs of RRV have been variably estimated, with earlier modeling studies suggesting approximately $15 million per year in disease-related costs (4), although more recent comprehensive analyses have estimated substantially higher annual economic impacts of up to $4.3 billion when accounting for medical expenditure, lost productivity, and indirect costs (53). These disease costs do not account for the substantial ongoing investment in mosquito surveillance and control programs, which in QLD alone exceeded $15 million annually by 2007 for treating approximately 40,000 hectares of coastal mosquito habitat (54). The combined burden of disease costs and prevention expenditure positions RRV as a significant public health and economic concern requiring sustained investment in surveillance, prediction, and control measures.

### Case definition

In Australia, notification of RRV infection requires laboratory evidence in line with the nationally adopted case definition. Since 1 January 2013, the revised case definition accounts for known cross-reactions of RRV IgM antibodies with other pathogens by requiring confirmatory evidence beyond a single IgM-positive result (55). A further revision implemented from 1 January 2016 removed single IgM positivity as a diagnostic criterion to improve specificity (55). Currently, a confirmed case is defined by virus isolation, detection of viral nucleic acid by PCR, or evidence of seroconversion or a significant rise in IgG antibody levels between paired sera. A probable case involves the detection of both RRV-specific IgM and IgG antibodies in a single specimen, unless IgG was detected more than 3 months prior. Both confirmed and probable cases are notified and form the basis of national surveillance (55).

### National overview and temporal patterns

**Fig 2:**
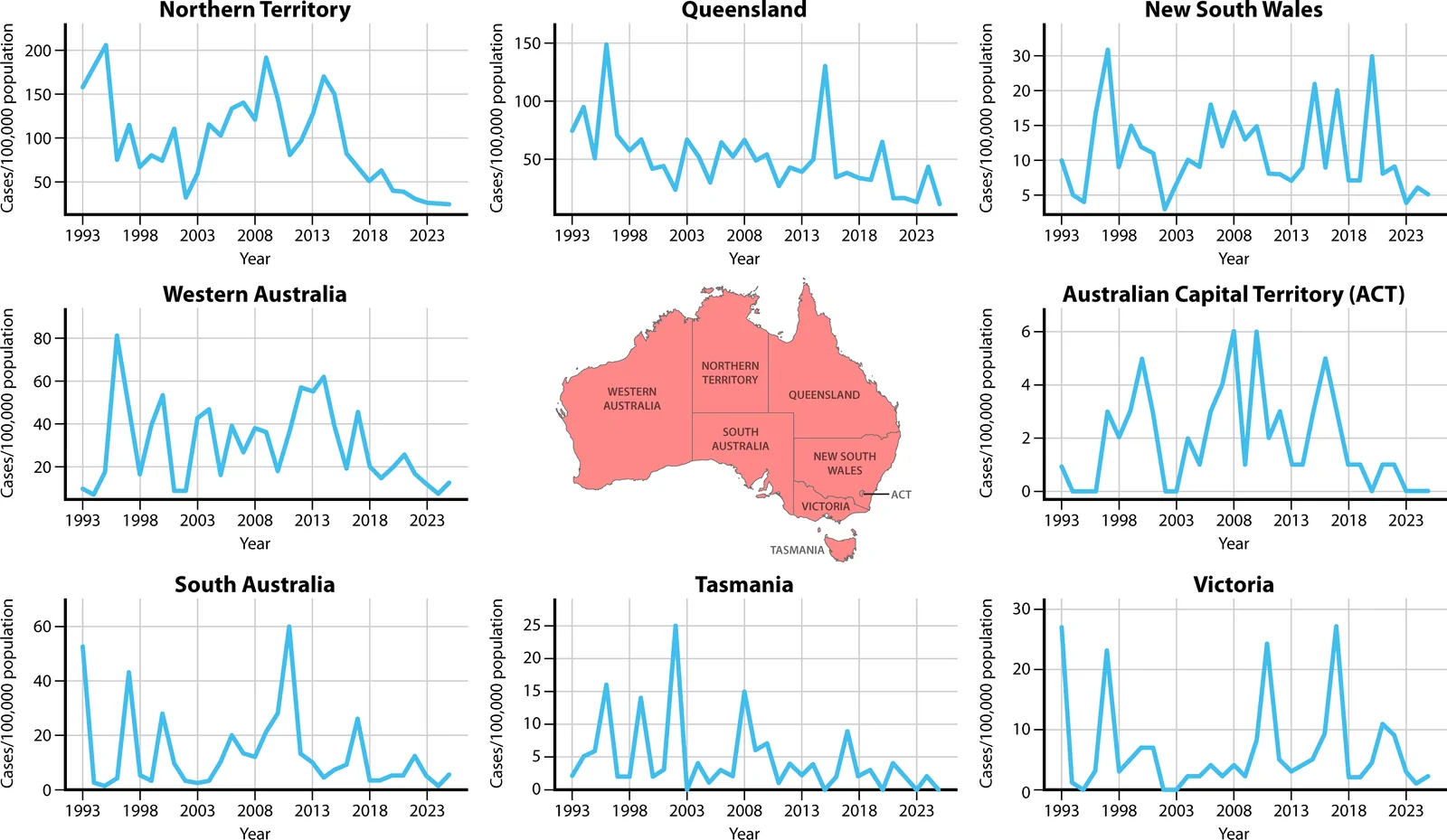
Ross River virus incidence (per 100,000) in Australia, 1993–2024 by state/territory. Source: Australian Government Department of Health (1). The base map was generated in R; the code uses rnaturalearth, specifically the ne_countries() function from the rnaturalearth and rnaturalearthdata R packages.

Australia experienced an annual range of 1,451–9,551 RRV notifications between 1993 and 2024, with a mean of 4,415 cases per year over this 35-year span. Trends since national notification began in 1993 show marked interannual cycling (Fig. 2), with major epidemic upswings roughly every 3–7 years, with major epidemics recorded in 1996 (7,765 notifications), 2008 (5,607 notifications), 2015 (9,532 notifications), and 2020 (6,327 notifications) (1). The average annual notifications increased by 31% for 2006–2015 compared to the previous decade (1996–2005) (1, 56), although more recently (2016–2024), average annual notifications decreased 29.7% (1), suggesting that notifications fluctuate around epidemic cycles rather than following a consistent upward trend. Notifications of RRV exhibit strong seasonality across most regions, with peak transmission typically occurring in late summer to autumn (February–April [57]), although notable exceptions have been documented, including the April–May peak observed during the 2020 outbreak in Brisbane, coinciding with COVID-19 lockdown measures (58). While surveillance data and research effort have been concentrated in Eastern Australia, where absolute case numbers are highest, northern regions of the country (such as the Northern Territory, northern Queensland, and the Kimberley region of Western Australia) experience substantially higher per capita notification rates and distinct transmission dynamics characterized by wet season rather than late summer peaks.

### Demographic patterns

Ross River virus infection affects all age groups but shows distinct demographic patterns. Analysis of national notification data (1) from 1993 to 2024 indicates a slight female predominance, with average annual incidence rates of 19.3 and 17.9 per 100,000 for females and males, respectively, based on population data from 2015 (female:male ratio, 1.09:1; Fig. 3) (1). This female predominance is most pronounced in the 15–59 year age group (female:male incidence ratio, 1.12:1), while older adults (≥60 years) show a male predominance (female:male incidence ratio, 0.80:1) (1), suggesting age-dependent differences in exposure, susceptibility, or access to medical services.

**Fig 3:**
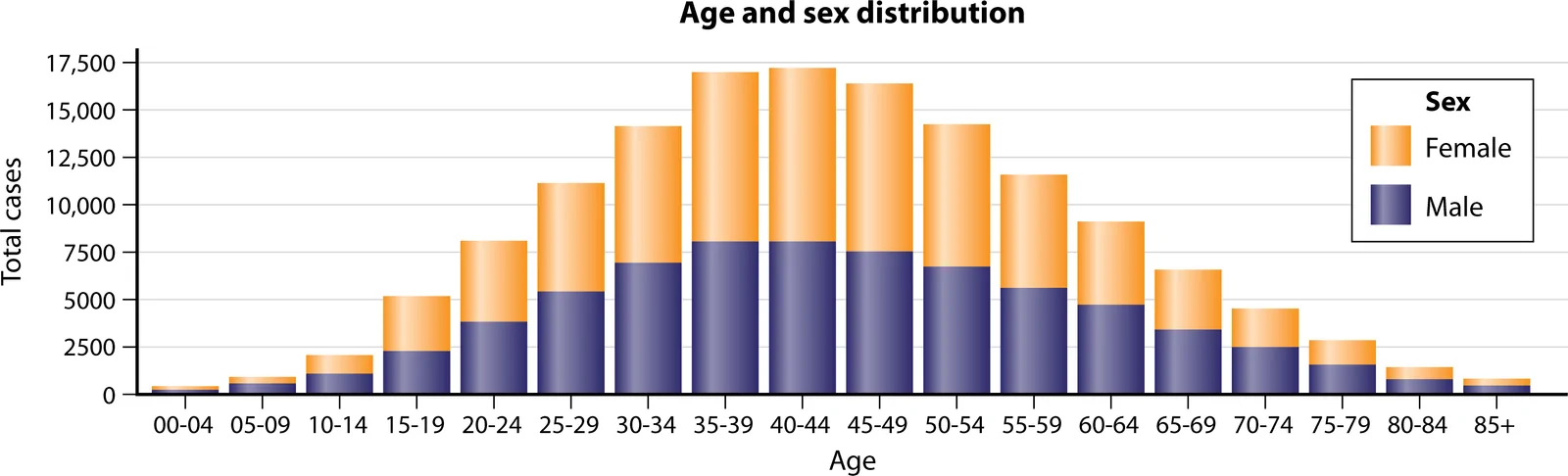
Sex distribution and age distribution of RRV notifications, 1993–2024. Source: Australian Government Department of Health (1).

Age-specific incidence analysis demonstrates that adults aged 40–44 years have the highest average annual incidence rate (32.4 per 100,000), followed by those aged 35–39 (33.1 per 100,000), 45–49 (32.0 per 100,000), and 30–34 years (24.7 per 100,000) over the 1993–2024 period (Fig. 3). Lower notification incidence rates are reported for children and elderly people, although clinical presentation and disease severity vary across age groups.

### High notification states: Queensland and New South Wales

Queensland consistently reports the largest number of cases among Australian states and territories, accounting for approximately 48% of all national notifications and contributing 43,699 total notifications between 2001 and 2020 (57), with an average annual incidence of 51 per 100,000 population. The 2014–2015 epidemic was particularly large, with QLD cases representing 63% of the national total and Brisbane alone accounting for 45% of the national case load (59) (Fig. 2). Northern QLD consistently experiences higher notification rates and incidence than southern QLD, with high-burden areas distributed along both coastal regions and western QLD. Specific areas, including Townsville, Mackay, Gladstone, Bundaberg, and the Sunshine Coast, exhibited both high absolute notifications and high cumulative incidence over 2001–2020 (57).

Spatial analyses of south-east QLD (2001–2016) identified 72 persistent hotspots (those that were hotspots for at least two individual years) predominantly at the rural-urban interface, where residential, agricultural, and conserved natural land use types intersect (56). Rural areas exhibited significantly higher incidence than urban areas (70 vs. 44 per 100,000; *P* < 0.001), with very few persistent hotspots centrally within major urban areas (56). This peri-urban pattern may reflect ecological requirements for sustained transmission. Peak transmission occurs between March and May, although recent outbreaks have exhibited extended or late season peak periods (57–59). Studies examining drivers of the two largest recent outbreaks (2014–2015 and 2020) identified distinct environmental and behavioral factors: the 2014–2015 epidemic followed unusually early and persistent rainfall (152% of average) after an exceptionally dry preceding year (55% of average) (59), while the 2020 outbreak was associated with COVID-19 lockdown behavior changes, including 80% increased time in green spaces and reduced travel distances, altering human-mosquito contact patterns (58).

New South Wales represents the second-highest burden state with distinct inland versus coastal epidemiological patterns and an average annual incidence rate of 11 per 100,000 population (1993–2024). Analysis of 1993–2013 data demonstrated that 64% of outbreaks occurred in inland regions, which also exhibited higher notification rates and antibody prevalence compared to coastal areas (60). The 2020 outbreak disproportionately affected northeast NSW, with the Hunter, New England, Mid North Coast, and Northern NSW regions accounting for 76% of the cases compared to a 5-year average for the same period (60). The 2020 outbreak in NSW followed a critical environmental sequence: 2 years of extreme drought (2018–2019), followed by substantial rainfall exceeding 200 mm coincident with high tides in February 2020 (60). Peak RRV notifications occurred 12 weeks after the high rainfall event, suggesting a predictive temporal cascade from environmental trigger to disease peak.

### Other states and territories

South Australia (SA) exhibits characteristic 3–4 year epidemic cycles with substantial disease burden during outbreak years (Fig. 2) and an average annual incidence rate of 13 per 100,000. The 2010–2011 outbreak was particularly severe with >1,400 cases. RRV in SA has strong seasonality, with 70% of cases occurring during summer and autumn months. Geographically, cases concentrate in southeastern regions along the River Murray and coastal areas, with the Riverland region identified as a persistent disease hotspot (61). Western Australia’s (WA) diverse climate zones, spanning the tropical Kimberley region, semi-arid Pilbara, and Mediterranean southwest exhibit fundamentally different outbreak drivers, with an average annual incidence rate of 29 per 100,000 population (1992–2024). Analysis across five sites during 1991–2014 identified minimum temperature as the most important universal predictor across all sites, although with varying lag periods (62). Northern tropical sites showed greater relative influence of minimum temperature compared to southern regions (62). Victoria has generally reported lower RRV cases compared to other states, with an average annual incidence of 6 per 100,000 population, contributing approximately 5% of national notifications in most years (Fig. 2) (1). However, during the major epidemics of 2010–2011 and 2016–2017, VIC accounted for up to 30% of national notifications during peak transmission months (1). The 2010–2011 epidemic produced 1,334 Victorian cases, representing 24% of the national total. Geographic hotspots concentrate in the Murray Valley region, Gippsland area, coastal regions, and inland waterways, while disease is not considered endemic in metropolitan Melbourne. Peak transmission occurs between March and April in this temperate climate, with the 2010–2011 outbreak following unusually wet summer conditions in areas characterized by typically hot, dry summers (63).

The Northern Territory (NT) has the highest incidence rate nationally at 96 per 100,000 (1993–2024), with endemic wet season transmission peaking December through March. The Darwin region averages 113 cases per 100,000 annually, with rural Aboriginal communities showing significantly elevated seropositive rates compared to urban non-Aboriginal residents (63). Despite the high per-capita rate, the NT’s relatively small population results in lower absolute case numbers compared with QLD and NSW. Tasmania had an average annual incidence rate of 5 per 100,000 (1993–2024) and experienced its first major RRV epidemic in 2002 with 117 cases, representing a 4-fold increase over previous annual averages (63). In contrast, the Australian Capital Territory (ACT) reports the nation’s lowest burden with an average annual incidence rate of 2 per 100,000 (1993–2024), representing only 0.1%–0.2% of the national total and averaging only 1–2 cases annually in recent years (2023–2024). This stark difference from surrounding NSW likely reflects ACT’s inland, elevated location lacking suitable coastal wetland habitats, a colder temperate climate, and reduced marsupial reservoir host populations, particularly in metropolitan Canberra (63).

### RRV risk to travelers and blood transfusion safety

Ross River virus presents a risk to travelers returning from endemic regions such as Australia, with infections reported in individuals who were diagnosed in non-endemic countries including Germany, the Netherlands, Singapore, and New Zealand (46, 64–67). While mosquito-borne transmission remains the primary route of infection, concerns regarding potential RRV transmission through blood transfusion have emerged, particularly during outbreak periods when asymptomatic viraemia in blood donors may occur (68–71). This has prompted increased surveillance and risk assessments within blood donation services in Australia, enhancing transfusion safety protocols. Although the overall risk to travelers and transfusion recipients is considered low, ongoing vigilance and preventative measures are essential to mitigate transmission (69, 70).

## ROSS RIVER VIRUS PHYLOGENETICS

The RRV single-stranded, positive-sense RNA genome is approximately 12 kb in length and encodes two open reading frames, ORF1 and ORF2, separated by a short non-coding region, which contains the sub-genomic promoter for ORF2 transcription (72). The genome is flanked by 5′ and 3′ untranslated regions (UTRs), terminating in a 5′ 7-methylguanosine cap and a 3′ polyadenylated tail. ORF1 encodes a non-structural polyprotein that is post-translationally cleaved to produce the individual non-structural proteins (nsPs), nsP1-4. ORF2 encodes the structural polyprotein, which is post-translationally cleaved into individual Capsid (C), Envelope glycoprotein 1 (E1), Envelope glycoprotein 2 (E2), Envelope glycoprotein 3 (E3), and 6K structural proteins (72, 73).

Phylogenetic investigations of RRV have evolved considerably over the past decades, with the development of and greater access to more robust sequencing methodologies. Past analyses, including small-scale outbreak investigations, have largely relied on partial genome sequencing, often of the E2 or nsP3 gene regions (59, 74, 75). In the first large-scale analysis, L. M. Sammels et al. (74) sequenced a 505 nt E2 region from 56 isolates, sampled between 1959 and 1991 from Australia and the PICTs. This study defined three genotypes, G1–G3, that had circulated over the sampling period. These genotypes, G1–G3, were designated the “northeastern,” “eastern,” and “western” genotypes, respectively, based on the apparent geographical demarcation of circulation. This classification has since been challenged with additional sampling, and the nomenclature and geographical demarcations are no longer valid (76).

The most comprehensive phylogenetic study to date employed whole genome sequence analysis of 106 RRV complete coding sequences, including 94 novel sequences derived for the study, sampled over a 59-year period (1959–2018) from throughout Australia and the PICTs (76). Before the publication of this genome-scale phylogeny, fewer than 10 complete RRV genomes were publicly available, largely derived from outbreak investigations and targeted evolutionary studies (77, 78). Four distinct genotypes were defined (G1–G4), including a newly described G4, the contemporary circulating lineage in Australia. G2 and G4 both formed distinct sub-lineages; G2A and G2B, and G4A and G4B. Bayesian analysis of the whole genome data set revealed novel lineage emergence roughly every decade over 50 years (76). Widespread shifts in genotype dominance were also observed, whereby an emergent lineage was sampled across broad geographical locations within a short period. A 12-amino acid duplication, first observed in the earliest PICTs isolate (F9073), was present in every sequenced G3 and G4 RRV, suggesting that it has become fixed in the population (79).

The only RRV isolate from Papua New Guinea, PNG3075, was collected from *An. farauti s.l*. in 1997 in the Western Province (40). Phylogenetic analysis showed PNG3075 formed a unique clade, distinct from all Australian and PICTs isolates, including the contemporary lineage, G4 (6). A timescale analysis dated the mean time to most recent common ancestor as July 1979, coinciding with the PICTs epidemic (April 1979–1980), suggesting that this RRV was introduced to PNG around that time and evolved locally, independent of Australian-derived RRV (6). Serological evidence indicates that RRV activity was present before the PICTs epidemic. This activity was likely caused by another Papua New Guinea lineage circulating before the introduction of the Papua New Guinea clade ancestor (37).

Advances in sequencing technologies have enabled the recovery of near-complete viral genomes directly from field-collected mosquitoes and clinical material. Meta-transcriptomics on large mosquito pools (up to 1,000 mosquitoes each) from rural VIC during heavy rainfall in 2016 detected RRV by RT-qPCR at all three locations: Mildura, Gannawarra, and Wellington (80). Three RRV consensus sequences, all typed as G4, were derived, with Gannawarra and Mildura isolates clustering in G4A, and Wellington in G4B (80). A tiling-amplicon sequencing approach was employed on an Oxford Nanopore Technologies platform to sequence 16 near-complete RRV genomes directly from whole-trap mosquito homogenates collected between 2000 and 2022 in coastal VIC (81). All genomes were assigned to the G4A lineage. Tiled-amplicon sequencing is a timely and cost-effective approach to deriving RRV genome sequences, without the need for cell-culture based isolation, with great promise for expanding the availability of RRV genome sequences for ongoing phylogenetic analyses.

**Fig 4:**
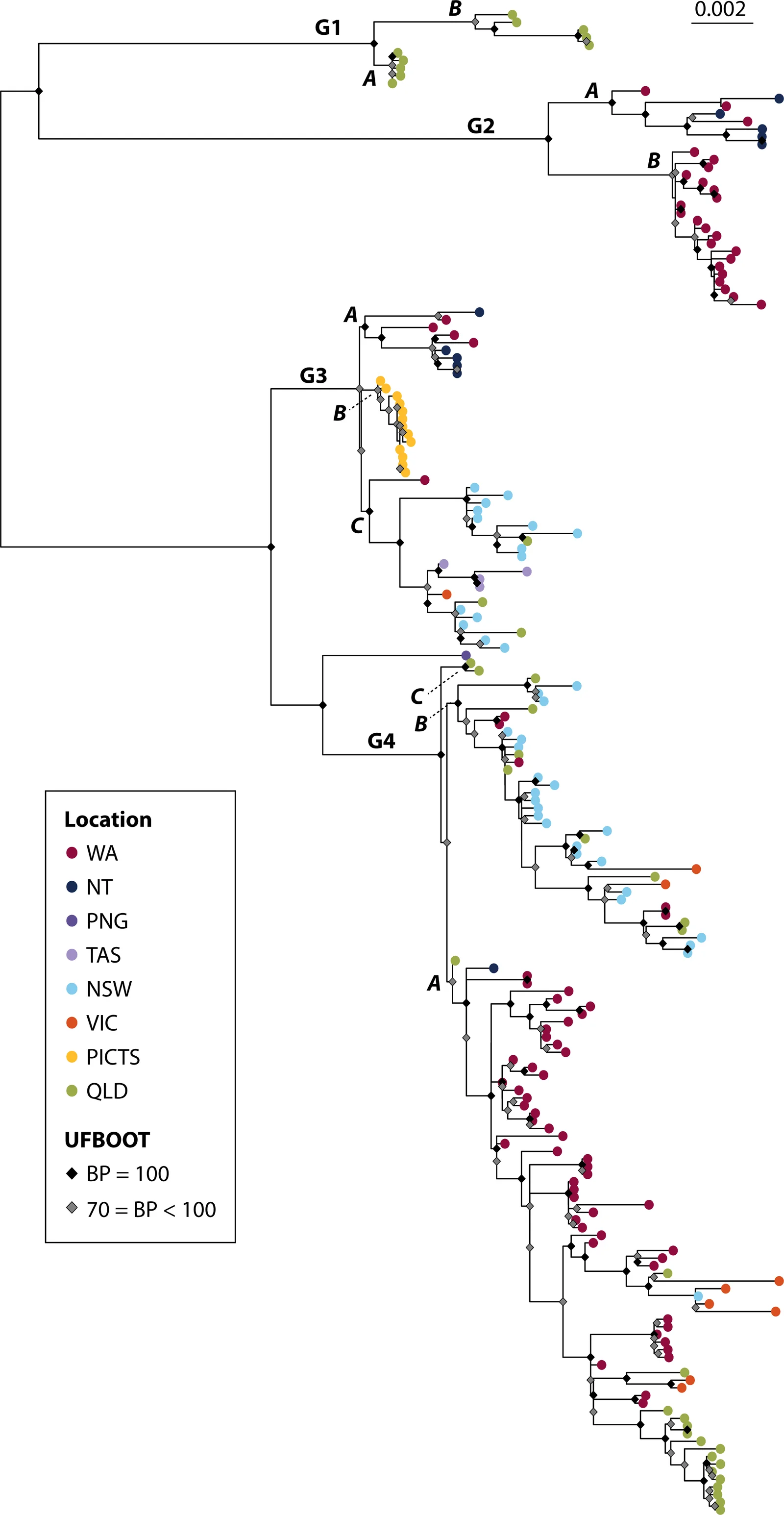
Re-constructed maximum-likelihood phylogeny of a 212 taxa near complete genome RRV data set. The taxa are summarized as circles at branch tips and are colored according to the broad location of sampling, as per the key. Nodes with strong bootstrap support values of >70% (1,000 ultrafast bootstrap replicates) are summarized on nodes by colored diamonds; black diamonds denote nodes with 100% bootstrap support, and gray diamonds denote bootstrap support of 70%–99%. The nucleotide substitution rate per site is represented by the scale bar. Genotypes and sub-lineages are labeled above their respective nodes. WA, Western Australia; NT, Northern Territory; PNG, Papua New Guinea; TAS, Tasmania; NSW, New South Wales; VIC, Victoria; PICTs, Pacific Island Countries and Territories; QLD, Queensland.

The increasing availability of RRV genome sequences has enabled finer-scale analyses of viral evolution and selection pressures (73); 79 RRV genome sequences were analyzed on a data set of 186 sequences, sampled over a 59-year period (1959–2018). All grouped within genotypes 1–4. Sub-lineages were defined within G1 (G1A and G1B) and G3 (G3A–C), which were previously defined as monophyletic clades. An additional sub-lineage within G4 (G4C) was also described with expanded sampling (73) (Fig. 4). Analysis with a similar 88 BFV genome data set revealed evidence of convergent evolution at sites within E3 and nsP1 associated with interactions with the host membrane and in the function of enzymes involved in viral replication. Selection pressure analysis found evidence of strong positive selection at nsP1 sites 248 and 441 (76). Both RRV and BFV had evidence of overall purifying selection pressure for nsP1 and E3, a likely consequence of replication in vertebrate and mosquito hosts (73, 77).

## INFECTION, IMMUNE RESPONSES, AND PATHOGENESIS

### RRV structure and replication

Transmission electron microscope (TEM) and cryogenic electron microscopy (cryo-EM) have resolved the structure of RRV at 15 Å (EMD-2965) (82) and 25 Å (83) and RRV bound to a neutralizing monoclonal antibody at 6.33 Å (PDB 6VYV) (84). These studies reveal that the structure of RRV is largely typical of the *Alphavirus* genus, forming an enveloped virion with T = 4 icosahedral symmetry and a diameter of approximately 70 nm (72). The host-derived lipid envelope contains 80 glycoprotein trimers, each composed of three E1–E2 heterodimers (83). The envelope incorporates host-derived cholesterol and sphingolipids, which are essential for maintaining envelope integrity and facilitating viral entry into cells. Beneath the glycoprotein envelope, 240 capsid proteins bound at the C-terminus of the E2 proteins form the nucleocapsid, which packages the viral RNA genome (83, 85).

RRV particles bind to host cells via one or more surface receptors or attachment factors, including Mxra8 (86, 87), CD147 protein complex (88), TIM-family proteins TIM-1/TIM-4/AXL (89, 90), and heparan sulfate if the RRV-E2 N218R substitution is present (91, 92). The subsequent steps in the replication cycle are probably similar to CHIKV (93). Briefly, receptor engagement triggers clathrin-mediated endocytosis, and low endosomal pH induces membrane fusion, releasing the viral RNA into the cytoplasm. Host ribosomes translate the nsPs, which assemble into replication complexes within “spherules” at the plasma membrane. These complexes produce both genomic and subgenomic RNA (sgRNA), with the latter translated into structural proteins. Capsid protein packages the genome to form nucleocapsids, while envelope proteins are processed through the endoplasmic reticulum and Golgi before incorporation into budding virions at the plasma membrane.

### RRV infection and interferon responses

**Fig 5:**
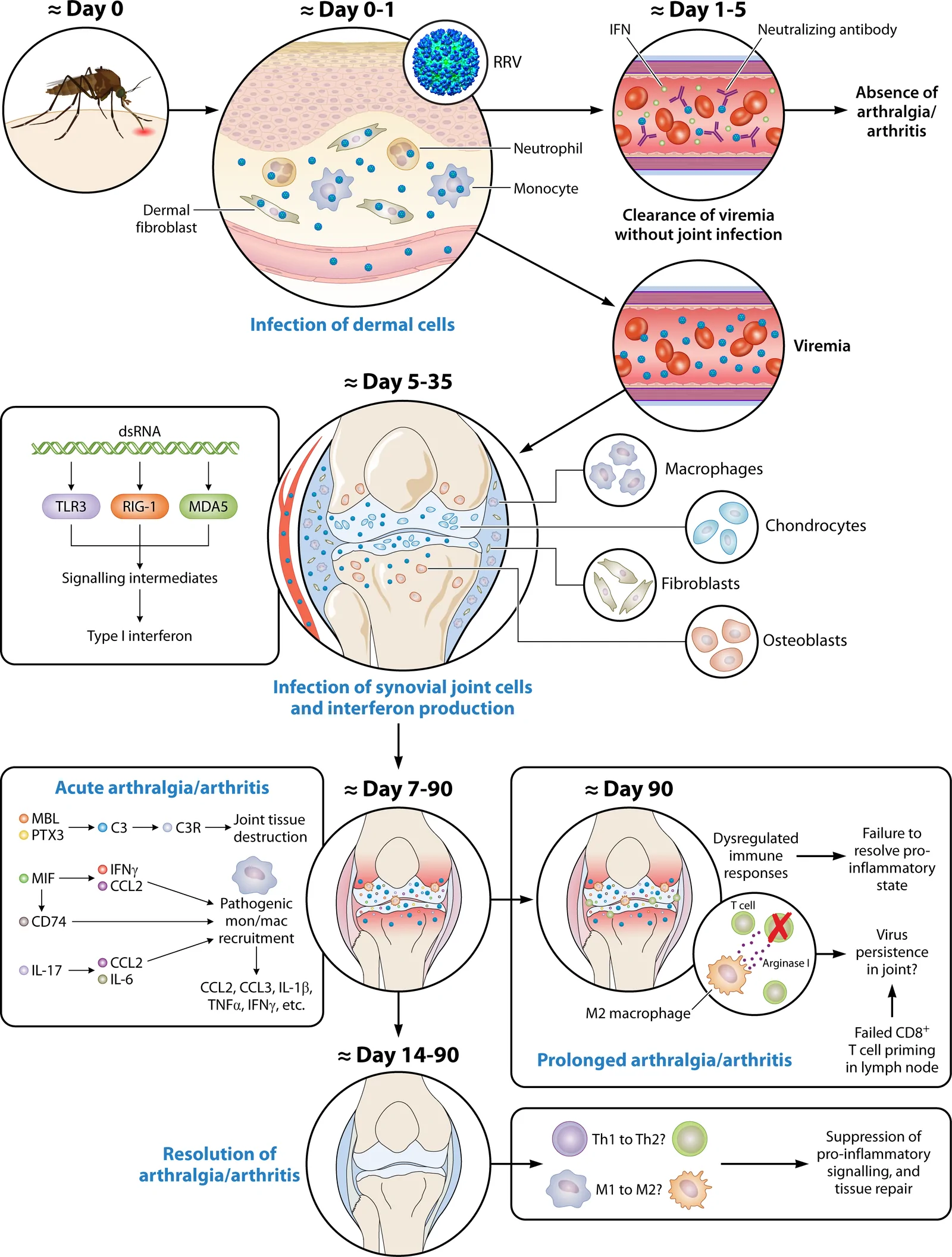
Ross River virus infection and immunopathological mechanisms of arthralgia/arthritis (©QIMR Berghofer). Following the bite of an infected mosquito, RRV replicates in dermal fibroblasts, monocytes, and neutrophils. The virus then enters the bloodstream, causing systemic viremia. Type I interferons (IFN) and neutralizing antibodies clear RRV from the circulation, and if this occurs before synovial joints are infected, arthralgia/arthritis may not develop (i.e., asymptomatic infection). RRV can disseminate to joints and replicates in synovial fibroblasts, macrophages, chondrocytes, and osteoblasts. Viral double-stranded RNA (dsRNA) replication intermediates are detected by Toll-like receptor 3 (TLR3), retinoic acid-inducible gene I (RIG-I), and/or melanoma differentiation-associated gene 5 (MDA5), triggering type I IFN responses. This initiates an inflammatory cascade characterized by infiltration of predominantly pro-inflammatory monocytes and macrophages, leading to arthralgia/arthritis. Mannose-binding lectin (MBL) and pentraxin-3 (PTX3) activate complement component 3 (C3), which signals through the C3 receptor (C3R) to promote joint tissue damage. Key pro-inflammatory mediators implicated in RRV-induced arthralgia/arthritis include macrophage migration inhibitory factor (MIF), interferon-γ (IFN-γ), monocyte chemoattractant protein-1 (MCP-1/CCL2), CCL3, CD74, interleukin-17 (IL-17), interleukin-6 (IL-6), interleukin-1β (IL-1β), and tumor necrosis factor-α (TNF-α). Resolution of RRV-induced arthralgia/arthritis likely requires viral clearance followed by a shift in immune cell polarization from pro-inflammatory Th1 to anti-inflammatory Th2 and regulatory T cells (Tregs), and from classically activated M1 macrophages to alternatively activated M2 macrophages, although the underlying sequence and timing of events remain unclear (denoted as “?”). Dysregulation of this polarization process may contribute to prolonged arthralgia/arthritis. Type I IFN signaling also restricts RRV replication in dendritic cells within draining lymph nodes, reducing the availability of viral antigen required for effective CD8^+^ T-cell priming. Arginase-1 further suppresses antiviral T-cell responses and may contribute to extended RRV infection and prolonged arthralgia/arthritis. (*Aedes vigilax* graphic is from reference 106, published under a CC BY 4.0 license.)

Following the bite of an infected mosquito, RRV replicates in dermal fibroblasts, monocytes, and neutrophils (Fig. 5), with infected monocytes and neutrophils also able to migrate to draining lymph nodes (94). The virus enters the bloodstream, leading to a systemic viremia, which is cleared by the type I interferon (IFN) and neutralizing antibody responses (95) by approximately day 5 post-infection in mouse models (96, 97). While it is unclear how closely this timeframe reflects human infection, viremia in humans typically resolves by the time symptoms appear (14). RRV subsequently infects cells within synovial joints, including fibroblasts (98), chondrocytes (99), osteoblasts (100, 101), and monocytes/macrophages (102) (Fig. 5). However, infection of hematopoietic cells is inefficient (103). Antibody-dependent enhancement (ADE) driven by sub-neutralizing titers of RRV IgG may permit the infection of monocytes and macrophages, as demonstrated *in vitro* (104, 105); however, its relevance to human disease remains unestablished.

Viral replication activates the type I IFN response through the detection of pathogen- and damage-associated molecular patterns (PAMPs and DAMPs) by receptors such as TLR3 (107); RIG-I/MDA5, which signal via MAVS (108); and other MAVS-independent pathways (109) (Fig. 5). Like many viruses, RRV evades the type I IFN response, with nsP, particularly nsP1, playing a key role in antagonism (108, 110–114). Additionally, N-linked glycans on the viral envelope influence IFN responses, with mosquito-derived RRV, which lacks high-mannose N-linked glycans, eliciting weaker IFN responses and allowing greater viral replication (94, 115, 116).

### Pathogenesis of RRV arthritis

Virus replication and the type I IFN responses trigger the inflammatory cascade that results in RRV arthritis. Monocytes and macrophages are the predominant immune cells infiltrating musculoskeletal tissues during disease (96, 102, 117, 118) and dominate synovial fluid in arthritic joints (102). Macrophage-derived proinflammatory mediators, such as monocyte chemoattractant protein-1 (MCP-1/CCL2), macrophage inflammatory protein-1 alpha (MIP-1α/CCL3), IL-1β, TNF-α, and IFN-γ, are key contributors to pathogenesis (119–121). Adaptive immune cells such as T and B lymphocytes play a minimal role in the development of RRV arthritis (96). Several proinflammatory pathways have been implicated in RRV pathogenesis. Despite its name, macrophage migration inhibitory factor (MIF) acts as a proinflammatory cytokine in RRV infection and is upregulated in the serum of RRV patients (122). MIF promotes arthritis by inducing IFN-γ and CCL2, which, together with the MIF receptor CD74, facilitate the recruitment of pathogenic monocytes and macrophages into infected joints (122, 123) (Fig. 5). Similarly, interleukin-17 (IL-17) is elevated in the serum of RRV patients and is produced by CD4^+^ and CD8^+^ T cells, neutrophils, and MHC class II^+^ macrophages, promoting arthritis by upregulating CCL2 and IL-6, leading to further infiltration of neutrophils and monocytes (124) (Fig. 5).

In addition to immune cells, infected non-immune cells also contribute to RRV pathogenesis. Osteoblasts can be productively infected by RRV and secrete IL-6, which contributes to bone loss (100), a disease process also observed in patients infected with CHIKV (125). Notably, individuals with pre-existing osteoarthritis may be at a greater risk of severe disease due to defective interferon responses in osteoblasts, resulting in increased RRV replication and cytokine production (101).

While earlier reports indicated normal serum levels of complement components C3 and C4 in RRV patients (14), recent studies have detected elevated levels of C3a in synovial fluid (118). Moreover, C3 and its receptor CR3 have been shown to drive joint tissue destruction independently of cellular inflammation in mouse models (118, 126), and this complement activation is largely dependent on the mannose-binding lectin (MBL) pathway (127, 128) (Fig. 5). Pentraxin 3 (PTX3) is associated with activation of the complement cascade and is elevated in RRV patients and contributes to pathogenesis (129).

### Resolution of RRV arthritis

In most cases, symptomatic RRV arthritis resolves within weeks. While research has focused on mechanisms of pathological RRV arthritis, very little is known about the mechanisms of RRV disease resolution. Thus, we have to draw most of our knowledge of RRV arthritis resolution from general principles of inflammation resolution (130). Once the virus is cleared from the joint by the inflammatory response, resolving the inflammation likely requires a complex interplay of immune mechanisms to restore tissue homeostasis. This process is regulated by pro-resolving mediators that shift the immune response from a pro-inflammatory to an anti-inflammatory state, gradually reducing pro-inflammatory cytokine levels in the joint until tissue homeostasis is restored (131). This involves a shift in immune cell polarization from pro-inflammatory Th1 to anti-inflammatory Th2 T cells and Tregs (132) and from classically activated M1 macrophages to alternatively activated M2 macrophages (130, 131) (Fig. 5). M1 macrophages have a pro-inflammatory phenotype and high antigen-presenting capabilities, key to the antiviral immune response. In contrast, M2 macrophages have an anti-inflammatory phenotype and high phagocytic activity, contributing to tissue repair and regeneration during wound healing. In a model of RRV myositis, resolution is marked by a shift from CD11b^hi^ Ly6C^hi^ pro-inflammatory monocytes to CD11b^hi^ Ly6C^lo^ CX_3_CR1^+^ macrophages that promote muscle repair (133). A comparable role in RRV arthritis resolution has not been demonstrated, although CX_3_CR1 expression is commonly associated with alternatively activated, tissue repair macrophage populations in diverse inflammatory and injury contexts (134, 135). During RRV arthritis, infiltrating immunosuppressive macrophages express high levels of Arginase I (a marker of M2 macrophages), which suppress antiviral T cell responses, delaying viral clearance and prolonging joint inflammation (136, 137) (Fig. 5). This highlights the need for precise timing in resolution signaling. If it begins too early, while the virus is still replicating, it can interfere with effective clearance and ultimately delay resolution, which may explain why some people have prolonged disease.

### Prolonged RRV arthritis

Some RRV patients can experience arthritis for several months. The reasons for variable disease duration are an active research area. Tappe et al. found that serum proinflammatory cytokines were higher in RRV patients with prolonged arthralgia (>30 days post-symptom onset) compared to both healthy controls and those with acute arthralgia (<30 days post-symptom onset) (138). The lack of inflammation resolution may be due to dysregulated immune responses leading to a persistent pro-inflammatory state or virus persistence in the joint (Fig. 5). There is very limited evidence of virus persistence in human clinical samples; 2 out of 12 sampled patients were positive for RRV nucleic acid by PCR in knee biopsy tissue at 5 weeks post-symptom onset (117), while another study identified RRV antigens in synovial monocytes and macrophages by immunofluorescence, but only in patients with samples taken 1–7 days post-symptom onset, while samples collected later in infection (at days 33, 58, and 94) were negative for RRV antigen (102). The related arthritogenic CHIKV, which causes more severe disease compared to RRV, has been detected in synovial macrophages and joint biopsies up to 36 months post-symptom onset by immunofluorescence (139, 140). However, replicating virus has not been detected in the joints of humans or animal models during chronic RRV or CHIKV disease, while viral RNA and proteins can be (141, 142), particularly within joint macrophages (143). Importantly, the reduction of viral RNA levels and inflammatory gene expression following antiviral treatment initiated during the chronic stage of CHIKV infection in mice demonstrates that residual viral RNA at day 28 post-infection reflects ongoing replication-competent virus, providing compelling evidence that viral persistence is a key driver of chronic inflammatory disease (143).

An immunological mechanism was posited in the previous review (14) which suggested that patients with chronic arthritis/arthralgia had defective cytotoxic CD8^+^ T cell responses, allowing viral persistence, while patients with rapid recovery from RRV disease had competent CD8^+^ T cell responses. CD8^+^ T cells infiltrate the synovial tissue of RRV patients (117), consistent with observations made in mouse models (96, 118). RRV-specific CD8 + cells from infected mice were able to lyse RRV-infected macrophages *in vitro* (144). RRV infection in mice induces a CD8^+^ T cell response that effectively clears virus from muscle tissue, but not from joints (145). Burrack et al. hypothesized that this may be due to either inefficient CD8^+^ T cell migration into joints and access to infected target cells and/or inflammation-driven suppression of T cell antiviral functions in the joint (145). The unexpectedly limited role of CD8^+^ T cells in clearing virus from the joints was further investigated, revealing that type I IFN signaling strongly restricted RRV infection in dendritic cells within the draining lymph node, thereby reducing the availability of viral antigen needed for effective CD8^+^ T cell priming (146) (Fig. 5). This provides further evidence for an immunological mechanism for RRV persistence in joint tissues, although convincing evidence of such persistence in clinical cases has yet to be demonstrated.

## RRV DIAGNOSIS AND DISEASE

### Laboratory diagnosis

Patients presenting with RRV disease have usually cleared viraemia, limiting the usefulness of direct detection diagnostic assays (RT-qPCR or antigen tests). For public health surveillance purposes, diagnosis for RRV in Australia is usually based on clinical and epidemiological information combined with IgM and IgG serology using enzyme-linked immunosorbent assay (ELISA) with commercially available kits (10). Clinicians also consider demographics, medical history including co-morbidities, and detailed exposure history to support diagnosis. Alphavirus-specific IgM generally appears around day 4 after symptom onset and remains detectable for 1–3 months (10). Seroconversion to neutralizing IgG usually occurs between days 4–10, and these antibodies appear to persist long-term, consistent with the absence of confirmed reinfection cases in the literature (10). A single serum sample is insufficient to confirm diagnosis, given a positive result could reflect a past infection or a false-positive cross-reaction with other alphaviruses. Careful attention is paid to differential diagnoses, including CHIKV (particularly in returning travelers), BFV, and other viral, bacterial, or autoimmune causes of arthritis, with testing for these differentials recommended where clinically appropriate (10). In the absence of virus neutralization assays (higher specificity, lower sensitivity), which are costly and impractical in routine clinical pathology, paired serology with a second serum sample collected at least 2 weeks after the first remains the gold standard for serological diagnosis (10) with a significant increase in IgG antibody titer between samples (typically > 4-fold) used to confirm recent infection (14). However, pathology providers often do not report numerical titers, as sera are commonly tested without a dilution series, precluding accurate titration (10, 147). Thus, confirmation of acute infection relies on paired serology demonstrating seroconversion (10, 147).

### Clinical presentation

Since the study of D. Harley et al. (14), the major advance has been in the documentation of progressive improvement in symptoms among patients without significant comorbidities, as shown in the studies by A. D. Mylonas et al. (148) and D. Harley et al. (149). RRV disease typically involves constitutional symptoms, rash, and rheumatic manifestations (150), with questionnaires identifying progressive improvement in rheumatic symptoms and overall health over 3–6 months among patients without comorbidities (148, 149). Subsequent reports describing European travelers who acquired RRV infection in Australia (64–66) presented a clinical picture consistent with earlier studies, with all patients experiencing arthralgia and four of six developing rash, and none displaying unusual symptoms or signs.

Two prospective studies of Australians with RRV disease have been published, focusing on changes in symptoms and signs over time (148, 149). D. Harley et al. (149) studied 47 patients through questionnaire data and clinical examination. Patients were reviewed on three occasions with mean time from symptom onset to first and third reviews being 1.1 and 3.6 months. The earliest and latest of the first and third reviews, respectively, were 0.2 and 6.5 months. The joints most involved with arthralgia at first review were, in decreasing frequency, ankles, wrists, knees, and interphalangeal joints of the fingers. The proportion of patients with symptoms progressively lessened through time, from 98% to 68%, 60% to 26%, and 53% to 11% at first and third reviews, for joint pain, myalgia, and joint swelling, respectively. Ordinal logistic regression showed progressive decline in the number of joint types involved. The study reported on rheumatological manifestations, and neither rash nor constitutional symptoms were included. A. D. Mylonas et al. (148) recruited 67 patients from the greater Brisbane area in southeast QLD and documented clinical status over 12 months using validated health questionnaires stratified by the presence of other conditions. At 6 months after specialist rheumatologist review, 28 had additional diagnoses, but only eight of these were rheumatic diseases. The three joints most often affected were the knees, wrists, and ankles. There was progressive health status improvement measured on SF-36 and CLINHAQ questionnaires for people without comorbidities, but the group with comorbidities had stable ill health between 3 and 12 months of follow-up. Together, these studies highlight that while most patients with RRV disease experience a gradual reduction in rheumatological symptoms over several months, recovery may be slower or incomplete in individuals with additional health conditions, highlighting the importance of personalized clinical management and long-term follow-up in affected individuals.

Data on RRV in vulnerable populations, such as immunosuppressed or pregnant individuals, are very limited. Available public health guidance consistently characterizes RRV as a non-fatal, self-limited febrile arthropathy with prolonged arthralgia in some patients, and fatal outcomes have not been reported. Among organ-transplant recipients (who typically have some degree of immunosuppression), RRV had no overt increased risk of hospitalization or death (151). A similar pattern has been reported for chikungunya (CHIKV) in transplant recipients, where it was suggested that the immunosuppression may if anything attenuate severe inflammatory sequelae (152). In pregnancy, a study conducted during the 1979 Fiji epidemic detected RRV-specific IgM in cord blood, consistent with *in utero* infection; however, all affected infants were clinically normal at birth (153). In the most severe case within a cohort of 96 RRV-infected patients, autoantibodies that neutralize IFN-α2 were detected (154), which suggests a defective type I IFN response may lead to increased viral loads and more severe disease.

## RRV PREVENTION AND CONTROL

### Medical interventions

A RRV vaccine, consisting of an inactivated whole-virus formulation with Alum (developed by Baxter BioScience), has demonstrated safety and immunogenicity in phase III human clinical trials (155). However, further commercial development has stalled due to the limited Australia-Pacific market, which reduces commercial viability. Renewed interest for a RRV vaccine may arise following the successful licensing of two CHIKV vaccines (live-attenuated IXCHIQ [156] and recombinant virus-like particle VIMKUNYA [157]), as well as advances in modern vaccine technologies that allow lower-cost “plug-and-play” approaches (158). The U.S. Food and Drug Administration (FDA) suspended IXCHIQ’s biologics license following post-marketing reports of chikungunya-like illness and encephalitis, including cases in which the vaccine-strain CHIKV was detected in cerebrospinal fluid (159). Pre-existing type I IFN-neutralizing autoantibodies have been implicated in permitting vaccine replication and neuroinvasion in affected older adults (160). The kinetics of infection, disease progression, and diagnosis (158) limit the window of opportunity for reducing viral replication using antiviral drugs (161, 162) or monoclonal antibodies (84, 95, 163). A phase I clinical trial of an mRNA-encoded monoclonal antibody against CHIKV (mRNA-1944) demonstrated a favorable safety and tolerability profile and induced sustained production of neutralizing antibodies (164). However, Moderna has since discontinued development of mRNA-1944, despite positive phase I results, choosing not to advance the candidate into phase II clinical trials. At present, there is no clinical development of a monoclonal antibody targeting RRV. As a result, clinical management has primarily focused on alleviating inflammatory arthritis and its associated symptoms. Non-steroidal anti-inflammatory drugs (NSAIDs) and paracetamol (acetaminophen) provide effective symptomatic relief for most RRV patients (14) and remain the first-line therapy. However, these treatments are insufficient for some patients (165), highlighting the need for more effective therapeutic options. Corticosteroids have been shown to improve outcomes in a small cohort of RRV patients (166); however, their potential adverse effects limit widespread use (14). Other immunosuppressive therapies, such as the disease-modifying antirheumatic drug (DMARD) methotrexate or anti-TNF agents (e.g., etanercept), show some efficacy in CHIKV patients after viral clearance and seroconversion. However, studies in mouse models indicate that administration before viral clearance and seroconversion can worsen RRV infection and disease (167, 168), and therefore, these drugs are not recommended during acute RRV disease. Pentosan polysulfate sodium (PPS) is a semisynthetic drug that mimics glycosaminoglycan and is used to treat a range of inflammatory diseases and non-infectious arthritis (169). PPS has been proven effective against RRV disease in preclinical mouse models (169) and in a phase 2a, randomized, double-blind, placebo-controlled clinical trial (170), and it is currently accessible for patients via the Therapeutic Goods Administration (TGA) Special Access Scheme.

### Control strategies targeting vectors

Due to the lack of a licensed vaccine or specific treatment for RRV infection, mosquito control and the promotion of personal protective measures are the main tools used to reduce disease incidence. Mosquito control, which is predominately undertaken by national, state/territory, and local governments, involves the suppression of mosquito populations to a level that virus transmission is interrupted. Unfortunately, broadscale mosquito control is often compromised by the different ecologies of vector species and the large geographical areas where they exist.

Physical habitat modification, such as the construction of drainage networks, filling or changing patterns of water movement can be used to eliminate ponding in swamps and marshes where larvae develop, thus providing a long-term control solution (171, 172). However, the potential environmental impacts of physically modifying open water bodies have restricted the use of this control strategy in recent years. Source reduction is another form of physical control that can be used to eliminate container habitats of species that undergo larval development in natural and artificial containers, such as *Ae. albopictus* and *Ae. polynesiensis* (173, 174), which are potential RRV vectors, particularly in PICTs (175).

Application of insecticides forms a major component of most mosquito control programs. Larval control of key RRV vectors involves treatment of larval habitats with the biorational larvicides *Bacillus thuringiensis* var. *israelensis*, a microbial insecticide, or *s*-methoprene, an insect growth regulator, via ground based or aerial application (176). Adulticiding is generally not used for routine mosquito control in Australia and is rarely undertaken in response to outbreaks of RRV. Applications of natural pyrethrums or pyrethroids via space sprays (fogging), or as residual surface spray, are occasionally used when mosquito populations have reached an action threshold. For instance, targeted adulticide application is applied during outbreaks of arboviruses, which potentially have a fatal disease outcome, like Murray Valley encephalitis or dengue viruses, or in response to incursions of exotic mosquito species (177–179).

Detection of elevated virus activity can prompt many jurisdictions to release public health messaging to encourage the public to avoid mosquito bites by practicing personal protective measures. Recommendations can include avoiding locations and times when mosquitoes are active, wearing long-sleeved loose-fitting clothing, ensuring insect screens in houses, caravans, and tents are fitted and in good repair, and the use of effective repellents. In Australia, repellents containing either DEET (*N*,*N*-diethyl-3-methylbenzamide), picaridin (2-(2-hydroxyethyl)−1-piperidinecarboxylic acid 1-methylpropyl ester) or oil of lemon eucalyptus (*p*-menthane-3,8-diol (PMD)) as the active ingredient are effective against key RRV vectors (180, 181) and are recommended for limiting exposure to mosquito bites (9).

## ECOLOGY OF RRV

Ross River virus has many vectors and hosts and can persist across diverse climatic regions and ecosystems. More than 80 species of vertebrates have been found to be naturally infected with RRV based on serological evidence, while RRV has been either detected in or isolated from at least 43 mosquito species. Fifty years of research indicate RRV ecology is not limited to a single taxonomic group of vectors and hosts. The persistence of virus across Pacific islands where marsupials do not occur, repeated isolation from horses, early isolates from birds, and experimental transmission demonstrated by multiple species (182) argue for a pluralistic, context-dependent framework recognizing that different species assemblages contribute to transmission in different ecological settings. Consequently, there are multiple RRV transmission cycles, which can be enzootic, epizootic, or cryptic and which are influenced by myriad biotic and abiotic factors, that can involve different species depending on climate, land use, and environmental conditions.

### Reservoir hosts

#### Criteria for reservoir host competence

Throughout this review, we distinguish between “hosts” and “reservoir hosts.” A host is any species that can be infected with RRV, as evidenced by seroconversion, viraemia, or virus isolation. A reservoir host, by contrast, is a species that sustains viral transmission by maintaining infection and serving as a source of virus for vectors. All reservoir hosts are hosts, but not all hosts are reservoirs; some species may be infected but fail to amplify or transmit the virus (so-called “dead-end hosts”).

The traditional framework for assessing reservoir host competence, established by G. Kuno and G.-J. J. Chang (183), includes three criteria: (i) virus isolation from animals under natural conditions, (ii) relatively high antibody prevalence in field-captured animals, and (iii) demonstration of viraemia of sufficient titer and duration to infect mosquitoes under controlled laboratory conditions. This definition focuses on intrinsic reservoir competence, that is, the physiological capacity to support viral replication and infect vectors, combined with evidence of natural exposure. However, a species’ contribution to ongoing transmission (i.e., its role as a reservoir host) also depends on ecological factors determining contact between hosts, vectors, and humans (182, 184). Ecological reservoir competence requires abundance and proximity to humans as well as physiological capacity. A reservoir host must have frequent contact with vector populations, be attractive to vectors as a blood meal source, be susceptible to infection, and produce sufficient viraemia to infect feeding vectors (11). Identifying reservoir host species therefore requires integrating experimental competence data with field measurements of host abundance, mosquito feeding preferences, and spatial ecology. The evidence for RRV reservoirs is summarized below using both traditional criteria and ecological context.

#### Evidence for vertebrate reservoir hosts: experimental infection studies

Between 1969 and 2001, seven experimental infection studies examined the competence for 18 vertebrate species using two viral strains: the prototype T48 (isolated from a human in Townsville in 1959) and B94/20 (isolated from *Culex annulirostris* collected in QLD in 1994) (185–191). Infection routes varied across studies and included via infected mosquito bite (*n* = 5 studies), subcutaneous injection (*n* = 2), and intravenous injection (*n* = 1) (191). Experimental infection studies typically employed small sample sizes (median = 9 individuals per species), and more than half simultaneously co-infected animals with other viruses including BFV, Murray Valley encephalitis virus, or Sindbis virus (182). The different methods used to infect animals and measure viraemia precludes direct quantitative comparisons across experiments.

#### Evidence for vertebrate reservoir hosts: field surveillance

**Fig 6:**
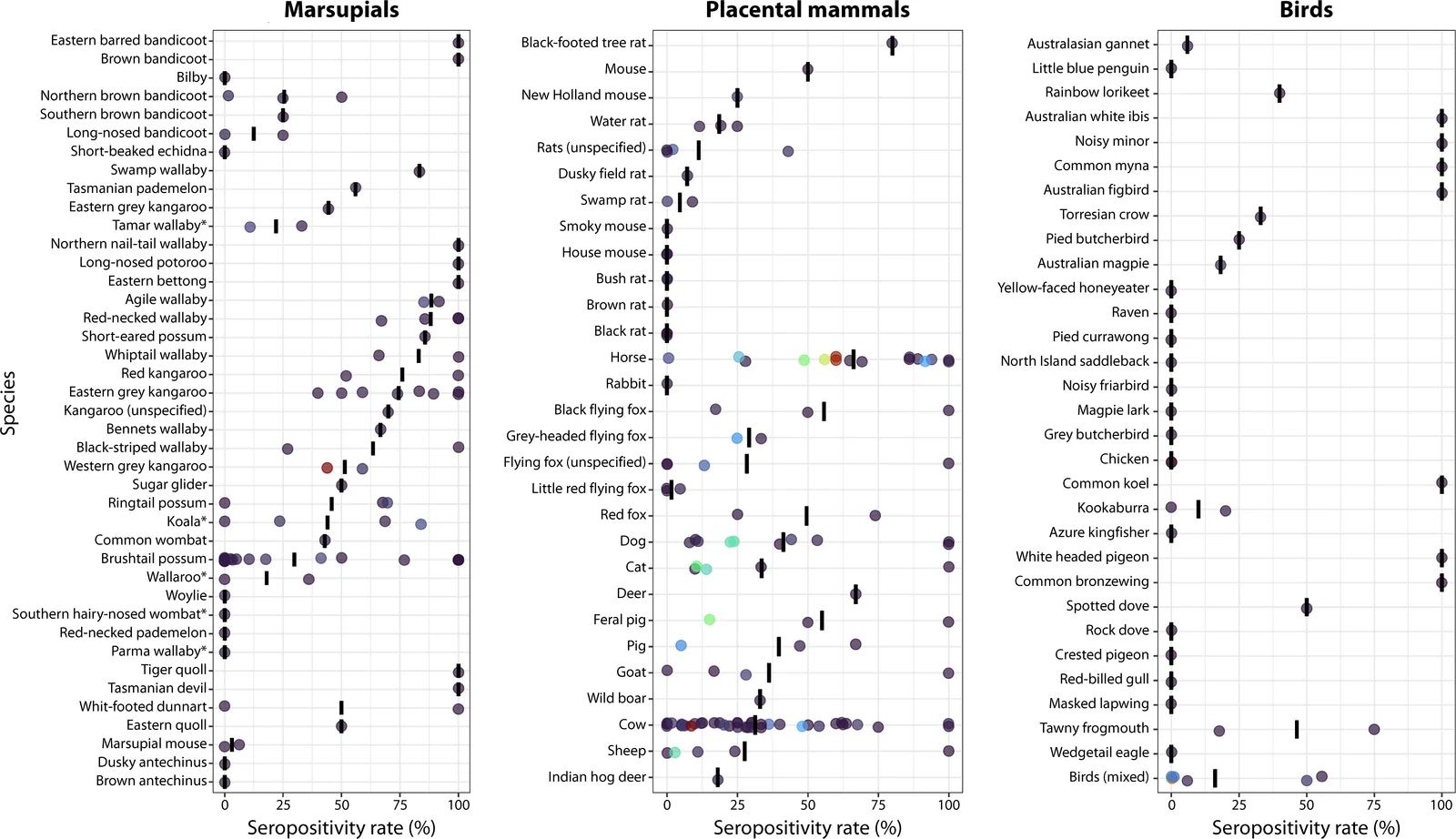
Scatter plots displaying the seropositivity rate (%) extracted from 38 published studies (Table S1) for each tested species, grouped into three panels: marsupials (left), placental mammals (center), and birds (right), organized by taxonomic groups. Each point represents a published or surveyed estimate of seropositivity, positioned along the horizontal axis by its reported rate and vertically by species. Dot color scales with the number of individuals tested per estimate, from blue (low, *n* < 100) to red (high, *N* > 750). Black dashed lines connect mean seropositivity values for each species where multiple data points are available. Those with (*) include studies utilizing zoo animals.

Experimental data are complemented by surveys looking for evidence of infection in field populations. The short duration of viremia reduces the likelihood of detecting virus or viral RNA during cross-sectional vertebrate surveys, which are usually restricted to a single time point. Thus, the virus has only been isolated from 20 individual animals: 15 from horses (192–194), 2 from agile wallabies, *Macropus agilis* (195), and 3 from passerine birds: magpie lark, *Grallina cyanoleuca,* flycatcher, *Myiagra rubecula*, and masked finch, *Poephila personata* (196). Horses are the only species besides humans known to exhibit clinical symptoms of RRV infection, including fever, lethargy, joint swelling, muscle soreness, and neurological signs (53). Thus, horses are potentially over-sampled because of frequent monitoring and veterinary attention relative to wildlife hosts. More than 35 serosurveys conducted between 1966 and 2024 tested >20,000 individuals from at least 100 species in Australia, New Zealand, and Fiji (Fig. 6; Table S1), with natural exposure to RRV found in at least 80 species. The results of serological studies need to be interpreted with some caution, as there can be cross-reactivity with other alphaviruses, particularly BFV, in immunological assays. Furthermore, while seroconversion to RRV demonstrates natural exposure from an infected mosquito (a necessary condition for a vertebrate species to contribute to transmission), seropositivity alone does not establish reservoir competence.

#### Marsupials

Marsupials are the most intensively studied group of potential RRV reservoir hosts. Experimental infections show they generally develop high, prolonged viraemia. Eastern gray kangaroos *Macropus giganteus* and brushtail possums produced some of the highest viraemias and can infect mosquitoes (182). Despite this, the single largest experimental infection study with 10 species and 92 individuals found no significant differences in viraemia duration or peak between marsupials, placental mammals, and birds, suggesting that variation occurs at the species rather than taxonomic group level (186). Virus isolation from two agile wallabies (of 17 tested) also provides compelling evidence for intrinsic reservoir competence of this species (195).

Serological studies reveal variation in RRV seroprevalence across marsupial species, locations, and time periods (Fig. 6; Table S1). Overall, seroprevalence is high in macropod species of varying sizes and ecological niches (Fig. 5), ranging from 27% (*n* = 11) in black-striped wallabies *Macropus dorsalis* (197) to 100% in Eastern gray *Macropus giganteus* and red kangaroos *Osphranter rufus* (*n* = 14 and 10, respectively) (197). Brushtail possums have displayed broad geographic variation in antibody prevalence, ranging from 0% (*n* = 72) in Sydney (198) to 77% in Tasmania (*n* = 13) (199), and 17%–41% in Brisbane surveys (*n* = 102 (200) and 182 (201), respectively). Ringtail possums *Pseudocheirus peregrinus*, historically poorly sampled, showed 70% (*n* = 145) seropositivity in a large 2020 South-East QLD study (201), suggesting that this species is an overlooked but potential reservoir host in urban areas; however, no experimental competence data exist. Koalas had a low reported seroprevalence across southern regions (0%–16%) (202, 203) and moderate-high values (68%–84%) (201, 204) in South-East QLD, where a longitudinal study documented age-related increases and active seroconversions, confirming recent local transmission (204). Smaller marsupial species, i.e., bandicoots, potoroos, and dasyurids, display variable seroprevalence (0%–100%; Fig. 6) but are often represented by small sample sizes, limiting generalization (182).

#### Placental mammals

The potential of placental mammals as reservoir hosts varies widely. Horses, the single most extensively surveyed vertebrate species (> 4,000 individuals tested), developed the highest recorded viraemia during which they infected 11% of *Cx. annulirostris* mosquitoes (182, 186). Serological surveys consistently demonstrate high seroprevalence in horses across diverse geographic settings, with values typically exceeding 60%. Early studies in coastal NSW reported 65% seroprevalence (205), while surveys in VIC found similar levels at 60% (206). More recent Australian surveys have revealed even higher exposure rates, reaching 86%–92% in northern QLD (207) and 94% in South-East QLD (201). This pattern extends beyond mainland Australia, with horses in Fiji showing universal seropositivity (52), confirming that RRV circulation among equine populations is both widespread and sustained across the geographic range of the virus. This evidence, combined with the 15 virus isolations from naturally infected individual horses (192–194), provides strong support for horses as competent reservoir hosts of RRV.

Pigs *Sus scrofa* and sheep *Ovis aries* demonstrated moderate intrinsic reservoir competence. They develop low-to-moderate viraemia sufficient to infect mosquitoes in laboratory conditions (187). Seroprevalence in pigs ranged from 5% to 50% (187, 195) in Australian studies and up to 67% in Fiji (52), while sheep have lower seroprevalence (0%–24%, *n* = 522; Fig. 5) (187, 197, 208). Cattle *Bos taurus* developed low viraemias, which suggest a limited reservoir competence, although seroprevalence ranged greatly, and cattle are frequently sampled due to historic sentinel surveillance for bluetongue virus (209) (Fig. 6). Collectively, the seroprevalence and low level viraemia suggest a role for cows as limited contributors to ongoing transmission. Dogs *Canis lupus familiaris* and cats *Felis catus* have reported low-to-moderate seroprevalence (Fig. 6) but fail to develop detectable viraemia after experimental infection and likely serve as dead-end hosts (189).

Of other placental mammals, low-to-moderate seroprevalence was shown in grey-headed flying foxes *Pteropus poliocephalus* and in black flying foxes *Pteropus alecto*, and low seroprevalence in little red flying foxes *Pteropus scapulatus* (Fig. 6). After experimental infection, viraemia was not detected in grey-headed flying foxes, although they were able to infect a small percentage (~3%) of feeding *Ae. vigilax* mosquitoes, suggesting that they may have potentially cryptic reservoir competence (190). Rodents generally have low seroprevalence (0% in house mice *Mus musculus*, 0%–19% in black rats *Rattus rattus*, 0% in bush rats *Rattus fuscipes*, and up to 9% in swamp rats *Rattus lutreolus*), with exceptions like New Holland mice *Pseudomys novaehollandiae* (25%), water rats *Hydromys chrysogaster* (12%–25%), and the black-footed tree rat *Mesembriomys gouldii,* which showed 80% seroprevalence in a small number of individuals sampled from the NT (210) (Fig. 4). However, experimental evidence of competence in rodents other than laboratory mice remains sparse or absent.

#### Birds

Birds have historically been considered relatively unimportant in RRV transmission cycles; however, the evidence presents a more nuanced picture. Experimental infections generate viraemia of lower magnitude and duration than in marsupials or placental mammals (182, 186). However, birds have demonstrated effective transmission of RRV to mosquitoes. Little corellas *Cacatua sanguinea* infected 14% of recipient *Cx. annulirostris*, an infection rate exceeding that of horses, despite the latter’s substantially higher viraemia (186). This discordance between viraemia magnitude and mosquito infection efficiency highlights that factors beyond viral load can influence susceptible vector infections. The earliest vertebrate RRV isolates were obtained from three passerine species (magpie lark, flycatcher, and masked finch) (196), confirming that at least some bird species develop detectable viraemia under natural conditions.

Birds have been less intensively sampled than mammals, with many early surveys testing mixed-species pools (182). Domestic chickens, *Gallus gallus domesticus,* appear to have poor reservoir competence, consistently showing very low or zero prevalence (0%–9% across studies; Fig. 6), consistent with experimental evidence of weak viraemic responses and poor antibody production (188).

Among wild native birds, serological evidence reveals patterns that may reflect ecological and behavioral differences rather than simple taxonomic divisions (Fig. 6). The highest seroprevalence has been documented in tawny frogmouths *Podargus strigoides* (18%–75%) (201, 211), a nocturnal, crepuscular species whose sedentary roosting behavior and temporal overlap with crepuscular-feeding mosquitoes may increase infection risk. Diurnal species show more heterogeneous patterns: some urban-adapted species like rainbow lorikeets *Trichoglossus moluccanus* (40%) and corvids (33%) demonstrate moderate-to-high exposure (201), while no serological evidence of infection in other common species has been found despite substantial sampling effort. This variation may reflect differences in habitat use, roosting ecology, mosquito host preference, or mosquito avoidance behavior rather than intrinsic physiological differences in susceptibility. Notably, historical surveys yielded strikingly different results, with northern QLD mixed-species samples showing 50%–56% seropositivity (*n* = 57) in one study (195), compared to just 0.4% in a much larger survey (*n* = 775, 104 species) (196) in the same region, suggesting strong spatiotemporal variation in avian exposure that complicates assessment of reservoir competence. However, the small sample sizes for most species (often *n* < 10) preclude robust statistical inference about interspecific differences in exposure risk or reservoir potential.

#### Humans

There is some evidence to suggest that humans may contribute as hosts to the transmission of RRV, particularly during outbreak events. This evidence includes the isolation of RRV from human serum (notably during the Cook Islands epidemic [32]); molecular detection of RRV RNA in blood donors, confirming viraemia of sufficient magnitude to infect susceptible mosquito vectors under natural conditions; and mathematical modeling that integrates human viraemia (measured at symptom onset) with mosquito infection and transmission probabilities to suggest that humans have moderate-to-high physiological competence (11). This host competence was comparable to the transmission potential of macropods in urban Brisbane due to the high abundance of people and their attractiveness in urban environments to competent vectors such as *Ae. vigilax* (11, 212). Despite these observations, the extent to which humans contribute to sustained interepidemic transmission remains uncertain, with overlapping confidence intervals in model estimates relative to established wildlife reservoirs and limited direct field data on human-mosquito contact dynamics.

### Mosquito vectors

Key criteria must be met for a mosquito species to be considered a vector of an arbovirus: (i) repeated detection of the virus in field populations; (ii) demonstrated ability for the mosquito to be able to become infected with and transmit the virus; and (iii) evidence that the species blood feeds on the vertebrate hosts of the virus (213, 214). There are over 350 species of mosquitoes in the Australasian region (215), but only a small proportion of these meet these criteria and are considered major vectors of RRV.

#### Virus detection in field populations of mosquitoes

Surveillance of RRV in mosquito populations typically involves collection of mosquitoes in CO_2_-baited light traps, pooling 10–100 individuals of the same species, and testing these pools for virus. Early detection methods relied on virus isolation using suckling mice, with identification via virus-specific antisera in hemagglutination inhibition, complement fixation, and/or neutralization tests (27, 216). The development of cell-culture-based systems, coupled with specific monoclonal antibodies in enzyme immunoassays (217) or immunofluorescence assays, facilitated the upscaling of mosquito processing in virus surveillance (218–220). More recently, virus-specific PCR-based assays (221) (with or without prior passage in cell lines) have enabled rapid detection from field samples (59, 222).

**Fig 7:**
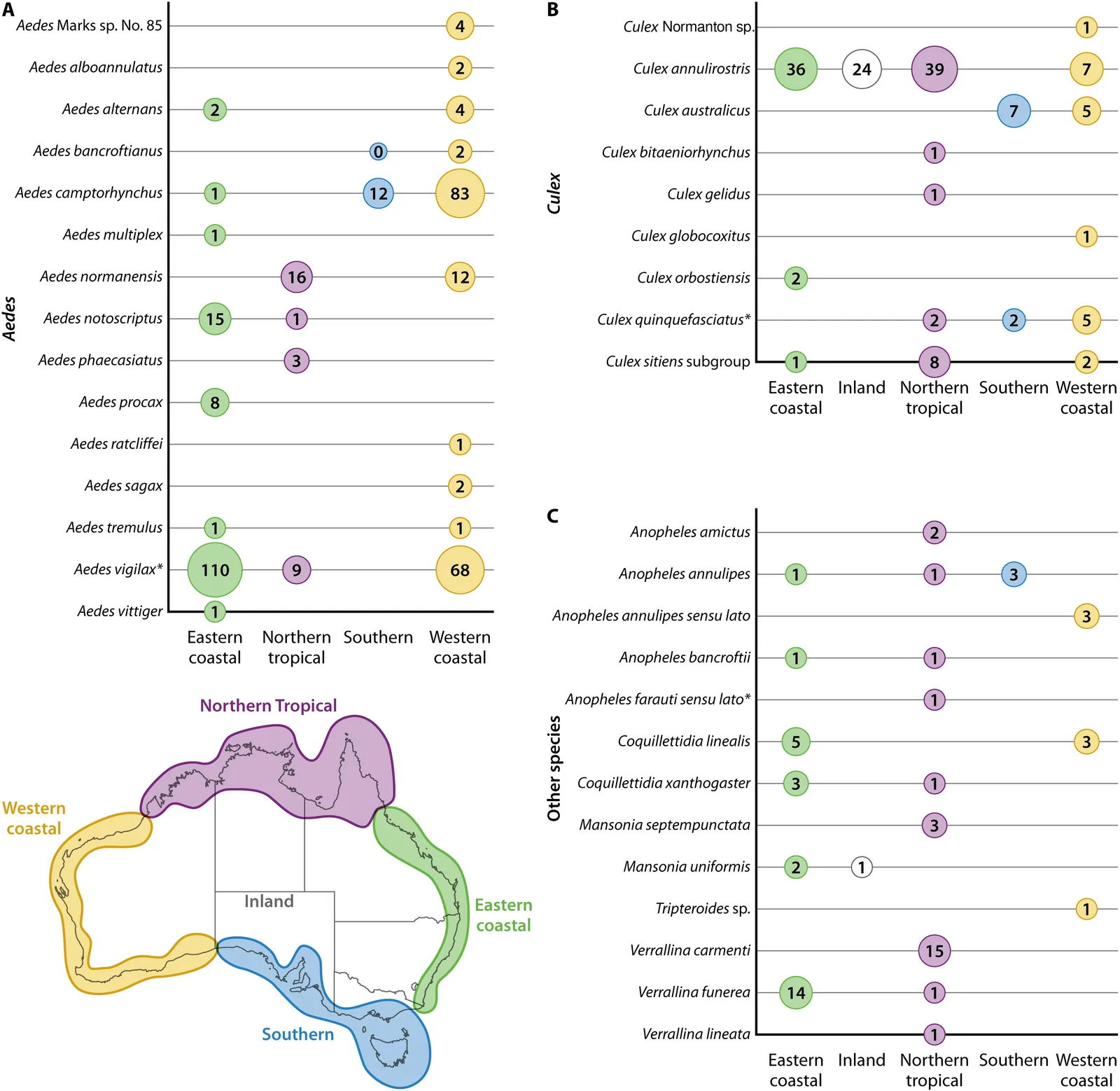
Panels A–C show RRV detection records by species (y-axis) and geographic region (x-axis) in Australia: (**A**) *Aedes* species, (**B**) *Culex* species, and (**C**) other genera, including *Anopheles, Coquillettidia, Mansonia,* and *Verrallina*. Each point indicates the number of virus detections for a species in a given region, with circle size proportional to the number of detections. Panel D displays the regional classification map used for data collation: eastern coastal, southern, northern tropical, western coastal, and inland regions. Data were extracted from 32 studies (Table S2). *Additional reports from the Pacific region not shown in the figure: *Ae. polynesiensis* (6 pools) from the Cook Islands; *Ae. vigilax* (3 pools) and *Cx. quinquefasciatus* (1 pool) from New Caledonia, and *An. farauti sensu lato* (1 pool) from Papua New Guinea.

Ross River virus has been detected in field-collected mosquitoes representing at least 43 species across six genera, with surveillance conducted across diverse Australian and Pacific settings (Fig. 7). Three species, *Ae. vigilax*, *Cx. annulirostris,* and *Ae. camptorhynchus*, together account for almost 70% of RRV detections reported in the literature. The geographic pattern of detections reflects underlying differences in mosquito ecology; saltmarsh *Aedes* species drive coastal transmission, whereas freshwater *Culex* populations are key inland vectors. However, this pattern should be interpreted cautiously, as sampling effort was higher in more populated areas, which are predominately located within 50 km of the coast. Furthermore, *Cx. annulirostris* may account for the majority of detections in inland areas, but RRV has also been detected in this species on numerous occasions in collections from coastal locations (218, 223, 224).

Limited surveillance in PICTs confirms RRV circulation in ecological contexts involving distinct vector communities from those in Australia. Collections from the Cook Islands yielded 6 detections of *Ae. polynesiensis* mosquitoes processed during the 1979–1980 epidemic (32), representing the first isolation from this endemic Pacific species. The virus has also been detected in *Ae. vigilax* (three detections) and *Cx. quinquefasciatus* (one detection) from New Caledonia surveyed during the late 1970s (225) and from a single pool of *An. farauti s.l*. collected from the Western Province of Papua New Guinea in 1997 (40). The disparity between the number of detections in the PICTs (11 total detections from 3 studies spanning 1981–2000) and the extensive Australian records (>730 detections from > 46 studies) potentially reflects profound differences in surveillance infrastructure and sampling effort, rather than transmission intensity.

#### Vector competence

The detection of virus in field-collected mosquitoes indicates which species are susceptible to infection but does not confirm whether they are able to transmit the virus. Vector competence experiments are conducted to assess the intrinsic ability of a mosquito species to become infected with and transmit an arbovirus. These experiments, undertaken under strictly controlled biological containment conditions, involve exposing cohorts of mosquitoes to an infectious blood meal, and after a period of incubation, testing their ability to transmit the virus. The key parameters derived from vector competence experiments are the infection rate (the proportion of mosquitoes infected with the virus) and the transmission rate (the proportion of mosquitoes that imbibed the infectious blood meal that transmitted the virus through their saliva).

**Fig 8:**
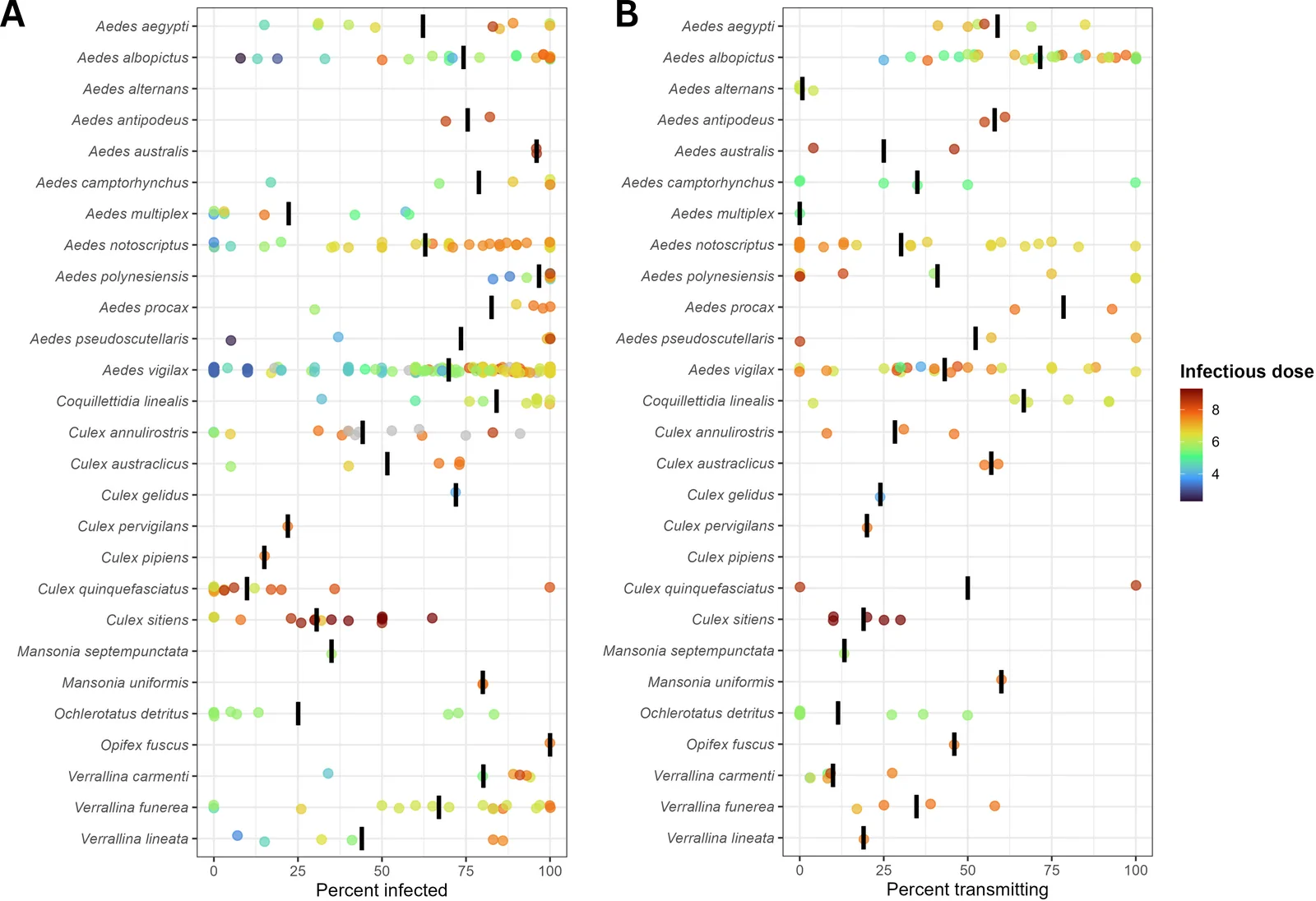
Experimental vector competence for RRV across Australian mosquito species. Panel **A** shows the percent of mosquitoes infected after exposure to RRV. Panel **B** shows the percent transmitting the virus. Each dot represents a separate experimental data point, colored by the infectious dose given to mosquitoes, and black ticks represent the mean for each species across all measures. Data were extracted from 40 studies (Table S3). *Ochlerotatus detritus* = *Aedes detritus*.

Experimental vector competence studies for RRV have been conducted on 27 mosquito species, with *Ae. vigilax* being the most studied species (Fig. 8). All species tested proved susceptible to RRV infection, and the majority could transmit the virus, although there was substantial interspecific and intraspecific variation in both susceptibility to infection and capacity for transmission. The large number of mosquito species shown to be competent laboratory vectors is not surprising, considering the diversity of species that RRV has been detected in during field studies.

Eight species (*Ae. aegypti*, *Ae. albopictus, Ae. antipodeus*, *Ae. procax*, *Ae. pseudoscutellaris*, *Cq. linealis*, *Cx. australicus,* and *Ma. uniformis*) demonstrated mean transmission rates above 50%, although *Ae. aegypti, Ae. albopictus,* and *Cq. linealis* are the only species in this group with more than two experimental data points. *Aedes procax* had the highest mean infection and transmission rates of 83% and 79%, respectively, followed by *Ae. albopictus* with the second highest mean transmission rate (72%), followed by *Cq. linealis* with a 67% transmission rate. *Ae. aegypti, Ae. antipodeus,* and *Cx. australicus* all had transmission rates between 57% and 58%, with the latter two estimates derived from only two data points, limiting confidence in these values.

The three species most frequently detected with RRV in field surveillance demonstrated moderate-to-high vector competence, although with notable variation between experiments (Fig. 8). Overall, *Ae. camptorhynchus* exhibited higher mean infection rates than *Ae. vigilax,* but a lower mean transmission rate (35%). *Ae. vigilax* showed an average infection rate of 70% and an average transmission rate of 43%, based on extensive laboratory data. However, transmission data for *Ae. camptorhynchus* were derived from only a single low-dose experiment, limiting comparisons of susceptibility to infection and subsequent transmission with other species. *Culex annulirostris* had mean infection and transmission rates of 44% and 28%, respectively, the lowest among these three species.

Several factors influence the outcomes of vector competence experiments. A threshold level of virus must be present in the infectious blood meal before mosquito infection and subsequent transmission occurs. Thus, there is a clear positive relationship between the infectious dose in the blood meal and both infection and transmission rates. For example, infection rates for *Ae. vigilax* increased from approximately 10% at low doses of 3.2 log_10_ infectious units (IU) of virus to 80%–100% at higher doses of 6–7 log_10_ IU (226). Similarly, only 17% infection was observed for *Ae. camptorhynchus* fed 4.6 log_10_ IU, but 89%–100% infection occurred in those fed 5.9–7.5 log_10_ IU (227).

The temperature that mosquitoes are incubated at can have a profound effect on vector competence. B. Kay and C. Jennings (228) demonstrated that while *Ae. vigilax* infection rates remained relatively high (60%–80%) in mosquitoes incubated at 18°C–32°C, the efficiency of dissemination through the hemocoel declined significantly at 32°C compared with 18°C–25°C. It was also shown that larval rearing temperature can influence vector competence, with mosquitoes reared at 25°C showing more consistent infection and dissemination patterns than those reared at 18°C or 32°C (228).

The time taken from when a mosquito imbibes the virus in an infectious blood meal to when it can transmit it is referred to as the extrinsic incubation period (EIP). The EIP is a critical value in virus-vector transmission dynamics, as a mosquito must survive the EIP, and the sooner a virus is transmitted, the higher the proportion of mosquitoes that will have survived to transmit the virus. Generally, as the EIP progresses, the proportion of mosquitoes transmitting the virus increases. For instance, after ingesting a blood meal containing 5.2 log_10_ IU of virus, transmission rates for *Ae. camptorhynchus* increased from 0% at days 3–4 to 25% at day 5, 35% at day 6, 50% at day 7, and 100% by day 9 (227). Some studies have demonstrated RRV transmission 3–4 days after mosquitoes imbibed an infectious blood meal (229, 230). The short EIP for *Ae. vigilax* in the B. Kay (230) study is ecologically significant, as this ephemeral species has a relatively short lifespan; hence, a higher proportion of mosquitoes could potentially survive to transmit the virus (231).

#### Incriminated mosquito vectors of RRV: reviewing the evidence

While numerous mosquito species are potentially involved in enzootic RRV transmission, large outbreaks are generally driven by elevated populations of key vectors, particularly *Ae. vigilax* and *Ae. camptorhynchus* in coastal areas and *Cx. annulirostris* in inland areas (222, 232, 233). In addition to being highly abundant during outbreaks, these species yield a relatively high proportion of detections, are competent laboratory vectors, and have host feeding patterns that provide contact between reservoir hosts and humans. The following section reviews the evidence incriminating mosquitoes as vectors of RRV and shows that in addition to the three key species, there are other species that could play a significant role in transmission cycles.

*Aedes vigilax* is the species with the most RRV detections from field collected mosquitoes (Fig. 7). *A. vigilax* is a competent laboratory vector, with a mean transmission rate of approximately 45%, but with up to 100% transmission reported in some experiments. Analysis of host feeding patterns has shown that *Ae. vigilax* obtains blood meals from a variety of hosts, including humans and those hosts implicated in RRV transmission (200, 234, 235). The larval habitats of this species are tidal saltmarsh and mangrove-associated brackish waters, with larval development triggered by inundation of vegetated upper marsh zones by higher than normal tides and rainfall (236, 237). As such, the geographic distribution of *Ae. vigilax* follows much of Australia’s coastline from the tropics through southwestern WA to temperate southern regions in VIC, with virus detection intensity greatest in coastal urban centers (Brisbane, Sydney, Perth) where saltmarsh habitat persists adjacent to human populations. Adults disperse 5–20 km inland from coastal larval habitats and up to 50 km wind assisted (238–240), defining the spatial extent of saltmarsh-associated transmission explaining the characteristic coastal concentration of human RRV cases in regions where this species dominates vector communities. In VIC, *Ae. vigilax* appears to have become established in the Gippsland and Wellington Shire Council areas (241, 242). Given that *Ae. vigilax* reaches peak abundance in the summer (243), this species may facilitate transmission in these locations during this time of the year when *Ae. camptorhynchus* populations are historically lower (241). This mirrors the situation in the Peel region of Western Australia, where *Ae. vigilax* became an important vector following the opening of the Dawesville Channel (233).

The southern Australian coastline harbors another coastal vector, *Ae. camptorhynchus*, that has yielded 138 RRV detections across 10 studies. In the only published vector competence experiments, *Ae. camptorhynchus* readily transmitted the virus to suckling mice (227), confirming its vector status. Like *Ae. vigilax*, a range of vertebrates serve as blood meal hosts for *Ae. camptorhynchus*, with marsupials accounting for 60% of blood meals in a study in WA (235, 244). The larvae of *Ae. camptorhynchus* inhabit pools in upper coastal saltmarsh zones subject to periodic inundation by extreme high tides and rainfall (236), although it has colonized inland areas affected by dryland salinity (245). Following inundation, populations of *Ae. camptorhynchus* rapidly increase, with adult emergence occurring 7–14 days post-flooding and dispersal up to 10–30 km from coastal larval habitats (246–248). This biology has been associated with temporal RRV outbreak patterns in southern Australia characterized by clusters of human cases appearing 6–10 weeks following extreme high tide events (62). Restricted to temperate and subtropical latitudes, this species' geographic range encompasses the areas of greatest disease burden in coastal regions of SA, VIC, southern WA, and TAS, where it drives the majority of temperate coastal transmission (233, 248, 249). Previously free of highly competent RRV vectors, the establishment of *Ae. camptorhynchus* in New Zealand in the late 1990s led to fears of a major RRV outbreak, particularly given the lack of immunity to the virus in the vertebrate host and human populations (250). A control program was implemented, which led to the eradication of the species after a 10-year battle and an investment of over $NZ70 million from the New Zealand government (250, 251).

In contrast to the coastal-restricted *Aedes* species, *Cx. annulirostris* exploits diverse freshwater habitats in both inland and coastal locations, including ephemeral ground pools, semi-permanent swamps, irrigation channels, and riverine floodplains (252). This plasticity in larval habitats allows *Cx. annulirostris* to occupy a large geographical area of Australia, where it has yielded RRV detections across inland habitats (Fig. 7) as diverse as pools associated with river systems (Murray-Darling, Fitzroy), irrigated agricultural regions (Darling Downs, Riverina), and urban freshwater wetlands (Brisbane, Cairns). The host feeding patterns of *Cx. annulirostris* are similar to *Ae. vigilax* and *Ae. camptorhynchus*, whereby it readily feeds on humans and vertebrates implicated as hosts of RRV (200, 234, 235). The species' capacity to rapidly colonize ephemeral flood waters following rainfall events resulting in adult population explosions underlies the observed association between flooding and RRV outbreaks in riverine and coastal regions (59, 253, 254). High relative abundance of *Cx. annulirostris* was associated with epidemic activity in inland and coastal areas of QLD and NSW during Australia’s largest recorded RRV outbreak of 2014–2015 (59, 255). Interestingly, in Brisbane during the 2014–2015 outbreak, *Ae. vigilax* populations were also higher than average, but peak abundance did not coincide spatially or temporally with human notifications, providing further evidence for the involvement of Cx. *annulirostris* as the main vector during the outbreak (59). High population densities could potentially offset the slightly lower vector competence observed in *Cx. annulirostris* compared with the other two key vectors.

Beyond the three species implicated above, RRV has been detected in a diverse assemblage of mosquitoes, and virtually all are able to transmit the virus in vector competence experiments, albeit at different rates. Of these species, *Aedes notoscriptus* has yielded 19 detections from six studies and is a moderately efficient laboratory vector. This species occupies a peridomestic niche, utilizing natural and artificial water-filled containers as larval habitats. B. H. Kay et al. (256) found this species to be one of the more abundant freshwater mosquitoes in Western Brisbane, QLD, and it readily feeds on a variety of hosts (200, 234, 235), including humans. This close association with humans makes *Ae. notoscriptus* a probable vector in urban and suburban environments.

*Aedes procax* is a flood water species distributed in coastal areas of NSW and southern QLD. While considered primarily a freshwater species, it is also found in brackish water sites that have been inundated by heavy rainfall (257). Limited data available for this species suggest that it feeds on a variety of mammals, including marsupials and humans (200, 234). Based on its high relative abundance, high vector competence (257), and field detections (223, 224), *Ae. procax* likely plays a role in RRV transmission, particularly after its larval habitats are flooded by above-average rainfall (257). Indeed, elevated populations of *Ae. procax* incriminated this species as a potential vector during the 2014–2015 RRV outbreak in Brisbane (59).

Following the southwest Pacific outbreaks between 1979 and 1981, four species of *Aedes (Stegomyia*) mosquitoes, *Ae. aegypti*, *Ae. albopictus*, *Ae. polynesiensis,* and *Ae. pseudoscutellaris*, were assessed for their vector competence for RRV (175, 258). All species had infection and transmission rates that did not differ greatly from established vectors in Australia, suggesting that they could play a role in virus transmission cycles. Of the species tested, RRV has only been detected in the field from *Ae. polynesiensis* (32), although lack of detection in the other species potentially represents limited sampling. The larval habitats of all these species are natural (coconut shells, tree holes, and leaf axils) and artificial containers (tyres, drums, and discarded household items) (259, 260). Humans account for a considerable proportion of blood meals taken by these species, particularly for *Ae. aegypti*, although feeding on other vertebrates does occur (234, 261, 262). Despite *Ae. albopictus* being present in countries in the Pacific for decades (263), this highly invasive species had not become established in Australia, despite being intercepted on numerous occasions at first ports of entry (264, 265). However, Australia has become more vulnerable to the establishment of *Ae. albopictus* since widespread populations were discovered in the Torres Strait in 2005 (266), prompting a suppression program that has managed to prevent the infestation from spreading to the mainland (179).

RRV has been detected in several species in the *Verrallina* genus, with detections primarily restricted to QLD, where populations of these mosquitoes can be locally abundant (190, 267). The three species in which RRV has been detected, *Ve. carmenti*, *Ve. lineata,* and *Ve. funerea*, are all capable of transmitting RRV in vector competence experiments, although transmission rates are variable between species and experiments (267, 268). Both *Ve. carmenti* and *Ve. lineata* are restricted to far north QLD, where they inhabit rainforest pools and shaded groundwater sites for larval development. In contrast, *Ve. funerea* has a widespread geographical distribution, ranging from northern NSW, through QLD and the NT and into Papua New Guinea, where it exploits fresh and brackish water swamps for larval development, especially those dominated by swamp she-oak (*Casuarina glauca*) and broad-leaved paperbark (*Melaleuca quinquenervia*) (269). While only a small number of these species have been analyzed for host feeding patterns, there is evidence that they will feed on humans and reservoir hosts of RRV.

Among *Culex* species, *Cx. gelidus* merits particular attention despite being a new addition to Australia’s mosquito fauna. A major arbovirus vector in Asia (270), *Cx. gelidus* was first discovered in 1999 in Brisbane, QLD (271). Since then, it has dramatically expanded its range, being collected from multiple locations in QLD, the NT, and, more recently, NSW (264, 272, 273). RRV has been isolated from this species in the field (218), and P. H. Johnson et al. (274) showed that its laboratory vector competence was not significantly different from *Ae. vigilax*. The larval habitats of this species are groundwater sites, and it proliferates in water with high organic content, such as sewerage settlement ponds or wastewater from animal processing plants (271, 272). Limited analyses of host feeding patterns of Australasian populations identified *Cx. gelidus* blood meals originating only from pigs and dogs (275, 276), mirroring the results of studies in Asia, which have shown that this species is primarily mammalophilic (277, 278).

There is less known about the role of other species in RRV transmission cycles. The virus has been detected in *Coquillettidia linealis* and various *Culex* species, including *Cx. sitiens* and *Cx. quinquefasciatus* from across multiple locations. Of these species, *Cq. linealis* is a highly competent laboratory vector of the virus (279) and an opportunistic blood feeder, readily feeding on marsupials and humans (234). This evidence indicates that *Cq. linealis* could be a RRV vector in locations where elevated populations are encountered and particularly where waterbodies with emergent vegetation are utilized as larval habitats (280). Despite multiple field detections, both *Cx. quinquefasciatus* and *Cx. sitiens* are relatively poor laboratory vectors of RRV (257, 281). They feed on a variety of hosts, but preferentially feed on birds (182, 212). Thus, these two species are likely to play only a limited role in RRV ecology.

#### Vertical transmission in mosquitoes

Vertical transmission from female to offspring, particularly via desiccation-resistant eggs oviposited by *Aedes* or *Verrallina* spp., provides a mechanism for the virus to survive periods of unfavorable environmental conditions (282, 283). Vertical transmission RRV has been directly demonstrated through the isolation of the virus from adult *Ae. camptorhynchus* mosquitoes reared from field-collected immatures in southeastern Australia and from adult males of *Ae. vigilax* and *Ae. tremulus* in northern WA, providing clear natural evidence of vertical transmission (220, 284). Experimental studies have confirmed vertical transmission in *Ae. vigilax* (230), although minimum infection rates were low. Vertical transmission may play a role in maintaining RRV during inter-epidemic periods when vertebrate hosts are scarce, thus contributing to the long-term persistence of the virus in mosquito populations. Ongoing surveillance and modeling have recognized vertical transmission as an important maintenance mechanism rather than a main driver of epidemics (284, 285).

## MODELING STUDIES OF RRV

Mathematical modeling of RRV has progressed from relatively simple climate-driven regressions and time-series analyses to a diverse set of approaches that included mechanistic transmission models and machine-learning methods. Collectively, these models have advanced understanding of RRV transmission by focusing on two central questions: what environmental and ecological factors drive RRV transmission, and how can these processes be harnessed to predict outbreaks with sufficient lead time for public health interventions?

### Modeling approaches

The earliest studies employed generalized linear models (GLMs), Poisson regression, and autoregressive time-series methods such as Autoregressive Integrated Moving Average (ARIMA) and Seasonal Autoregressive Integrated Moving Average (SARIMA) models to link weather conditions with RRV notifications (61, 286–290). These sought to statistically link weather conditions (including rainfall, temperature, humidity, and tidal data) with RRV notifications. These models often incorporated lagged variables to capture delayed effects of environmental factors and used Poisson or negative binomial error structures to account for overdispersion. Spatial models were used to detect clustering and autocorrelation across RRV notifications (291, 292), including approaches such as Bayesian spatio-temporal models developed by W. Hu et al. (293), which integrated spatial variation with temporal trends across QLD. Logistic regression models applied in the Murray River region predicted epidemic occurrence with 70%–90% accuracy, particularly when mosquito surveillance data were included (294, 295).

Mechanistic approaches shifted the focus from statistical correlation to simulating biological and ecological processes underpinning transmission. For example, K. Glass (296) developed stochastic SEIR models accounting for mosquito–host interaction dynamics, while S. Carver et al. (247) and I. Koolhof and S. Carver (297) used simulation frameworks to examine the contribution of host abundance and competence in transmission cycles. M. P. Kain et al. (298) introduced a next-generation matrix framework that quantified the role of individual hosts and vectors in Brisbane, showing that although macropods were highly competent, humans and birds were more important in sustaining urban transmission. Complementing this, I. Koolhof et al. (62) fitted deterministic SIR models to surveillance data from eight epidemic centers, demonstrating that both macropods and possums were required to explain observed dynamics. Broad-scale comparative risk assessments have also been deployed; for instance, a comparative risk assessment framework (3) modeled the effect of climate change on RRV-associated years lived with disability between 2003 and 2018 and attributed 19.1% to rising temperatures. Additionally, J. A. Tall and M. L. Gatton (253) used generalized estimating equations to show that riverine flooding increased outbreak risk in inland NSW, emphasizing hydrological factors as critical transmission drivers.

Forecasting studies have increasingly used advanced regression and machine learning methods. Negative binomial regression models have been applied to predict outbreaks in VIC and QLD, achieving predictive accuracies between 64% and 81% (299–302). At the national scale, M. Sakib and T. Siddiqui (303) developed an ensemble deep learning model based on long short-term memory networks, which outperformed ARIMA and other neural network approaches when predicting case counts from surveillance data. Although machine learning models provide high forecasting accuracy, they tend to offer less insight into transmission mechanisms, limiting interpretability for control programs.

### Predictors

Environmental drivers such as rainfall, temperature, humidity, and tidal height remain the most frequently employed and robust predictors of RRV transmission risk. Rainfall, temperature, humidity, and tidal height were repeatedly associated with incidence in early models (286, 289, 304). River flow and flooding were significant predictors in inland systems (253, 290). More recent studies incorporated vegetation indices (e.g., NDVI), land cover classifications, human footprint, and water bodies as proxies for habitat suitability (302, 305–307). Using a mechanistic modeling approach, M. S. Shocket et al. (12) identified a nonlinear effect of temperature on RRV transmission. Using laboratory data from vector species, they found that transmission of RRV peaks at 26.4°C and declines below 17.0°C and above 31.5°C. This thermal optimum explained broad geographic and seasonal patterns of RRV, with year-round transmission in tropical areas and seasonal outbreaks in temperate regions. The model predicts that warming may increase transmission risk in temperate zones where most Australians live but reduce it in already warm tropical areas.

Mosquito abundance, when available, improved predictive performance substantially. For example, in Brisbane, mosquito density explained up to 83% of variation in RRV cases (288), while in WA, integrating mosquito surveillance increased model sensitivity for outbreak detection from 64% to 90% (295). Studies such as L. J. Walker et al. (308) showed that mosquito abundance and virus-positive pools were strong predictors of human cases. Although host data have been underrepresented in predictive models, largely due to limited availability of detailed abundance and competence data spatially and temporally, mechanistic modeling consistently underscores the critical role of hosts in sustaining RRV transmission. For example, M. P. Kain et al. (11) showed the role of birds and humans in Brisbane transmission, while I. S. Koolhof et al. (285) found possums and macropods were necessary across epidemic centers, and another model found kangaroo counts improved lagged regression models in NSW (305). This growing body of evidence supports the notion that RRV transmission involves complex, multi-host systems that vary across ecological contexts. Additionally, socio-economic and demographic variables, including remoteness, population density, and indices of socio-economic disadvantage, have been incorporated with spatial models to better represent human factors modulating exposure risk and transmission dynamics (293, 302, 309). Holistically integrating these host and human population factors with environmental and entomological data remains a critical frontier for advancing RRV predictive modeling.

### Model performance and implementation

Model performance metrics vary across modeling frameworks and data contexts. SARIMA and Poisson models explained more than 70% of variation in RRV notifications in Brisbane, reflecting the strong seasonality and environmental drivers (304). Negative binomial models achieved 64%–81% accuracy in predicting case counts in VIC and QLD, with specificities often above 90% but lower sensitivities for outbreak detection, indicating challenges in early warning (299–301). Spatial and Bayesian spatio-temporal models techniques improved the identification of high-risk areas and improved fit, but validation was often limited by short time series (293). Mechanistic models provided explanatory insights into host and vector roles but generally did not predict cases directly. At the population level, Y. T. Damtew et al. (3) quantified the temperature-attributable burden, while W. Qian et al. (302) showed that including recent cases improved predictions compared to pooled models. Machine learning achieved the highest forecasting accuracy, with M. Sakib and T. Siddiqui (303) (2023) ensemble Long Short-Term Memory model reporting a normalized RMSE of 0.15. Machine learning models provide high accuracy but little insight into mechanisms, which constrains their interpretability for control programs.

Despite the significant research advances, operational use of RRV models remains limited. The Victorian Outbreak Surveillance System, which integrates regression models with climate and mosquito monitoring, is the only established public health application (300). Other models remain research-focused, limited by underreporting of case data, variable mosquito, host, and virus surveillance, and variability in the effects of environmental predictors across locations.

## CONCLUSION

When Harley et al. (14) published the last cross-disciplinary review of RRV 25 years ago, they highlighted key areas for future research, including further study of the vectors and vertebrate hosts posing risks to humans; investigation of environmental and behavioral determinants of infection; and basic research on immunology and pathology to improve treatment. Since then, many of these areas have been substantially advanced. This review has synthesized those advances across virology, immunology, ecology, entomology, epidemiology, and environmental science to provide an integrated understanding of RRV transmission and disease. Building on this integrated synthesis, the path forward for research and public health efforts involves several key priorities: (i) enhancing integrated surveillance of human cases, mosquito vectors, reservoir hosts, and viral genetics through harmonized methods and improved data sharing across Australia and the Pacific, with particular attention to underreported populations, including Indigenous Australians and remote communities where the true burden of diseases is likely underestimated; (ii) improved understanding of the relation between notifications, infections, and disease; (iii) expanding translational research to advance the development of better diagnostics, vaccines, antivirals, and immunomodulatory therapies for those at risk of prolonged illness; (iv) embedding climate-informed predictive models within public health practice to enable timely, locally adapted vector control and risk communication that considers changing land use, urban growth, and vulnerable populations; and (v) adopting a One Health approach that integrates ecological, veterinary, and human health interventions to reduce the current burden and prepare for the increasing threat posed by RRV in a changing Australia-Pacific region. Addressing these priorities is essential to reduce the significant and ongoing public health, social, and economic impacts of RRV infection, enhance outbreak preparedness, and mitigate the growing risks posed by environmental change across Australia and the Pacific region.

## SUPPLEMENTAL MATERIAL

Supplemental tables cmr.00099-25-s0001.xlsx Tables S1 to S3.

ASM does not own the copyrights to Supplemental Material that may be linked to, or accessed through, an article. The authors have granted ASM a non-exclusive, world-wide license to publish the Supplemental Material files. Please contact the corresponding author directly for reuse.
